# Label-Free Physical Techniques and Methodologies for Proteins Detection in Microfluidic Biosensor Structures

**DOI:** 10.3390/biomedicines10020207

**Published:** 2022-01-18

**Authors:** Georgii Konoplev, Darina Agafonova, Liubov Bakhchova, Nikolay Mukhin, Marharyta Kurachkina, Marc-Peter Schmidt, Nikolay Verlov, Alexander Sidorov, Aleksandr Oseev, Oksana Stepanova, Andrey Kozyrev, Alexander Dmitriev, Soeren Hirsch

**Affiliations:** 1 Faculty of Electronics, Saint Petersburg Electrotechnical University “LETI”, 197376 Saint Petersburg, Russia; dsagafonova@etu.ru (D.A.); aisidorov@etu.ru (A.S.); osstepanova@etu.ru (O.S.); abkozyrev@etu.ru (A.K.); 2 Institute for Automation Technology, Otto-von-Guericke-University Magdeburg, 39106 Magdeburg, Germany; liubov.bakhchova@ovgu.de; 3 Department of Engineering, University of Applied Sciences Brandenburg, 14770 Brandenburg an der Havel, Germany; marharyta.kurachkina@th-brandenburg.de (M.K.); soeren.hirsch@th-brandenburg.de (S.H.); 4 Faculty of Electrical Engineering, University of Applied Sciences Dresden, 01069 Dresden, Germany; marc-peter.schmidt@htw-dresden.de; 5 Molecular and Radiation Biophysics Division, Petersburg Nuclear Physics Institute Named by B.P. Konstantinov, National Research Centre Kurchatov Institute, 188300 Gatchina, Russia; Verlov_NA@pnpi.nrcki.ru; 6 Fuculty of Photonics, ITMO University, 197101 Saint Petersburg, Russia; 7 FEMTO-ST Institute, CNRS UMR-6174, University Bourgogne Franche-Comté, 25000 Besançon, France; aleksandr.oseev@femto-st.fr; 8 Department of Ecological Physiology, Federal State Budgetary Scientific Institution “Institute of Experimental Medicine” (FSBSI “IEM”), 197376 Saint Petersburg, Russia; dmitriev.av@iemspb.ru

**Keywords:** label-free biosensor, microfluidics, proteins detection, optical biosensors, impedance spectroscopy, plasmon resonance, point-of-care, diagnostic devices

## Abstract

Proteins in biological fluids (blood, urine, cerebrospinal fluid) are important biomarkers of various pathological conditions. Protein biomarkers detection and quantification have been proven to be an indispensable diagnostic tool in clinical practice. There is a growing tendency towards using portable diagnostic biosensor devices for point-of-care (POC) analysis based on microfluidic technology as an alternative to conventional laboratory protein assays. In contrast to universally accepted analytical methods involving protein labeling, label-free approaches often allow the development of biosensors with minimal requirements for sample preparation by omitting expensive labelling reagents. The aim of the present work is to review the variety of physical label-free techniques of protein detection and characterization which are suitable for application in micro-fluidic structures and analyze the technological and material aspects of label-free biosensors that implement these methods. The most widely used optical and impedance spectroscopy techniques: absorption, fluorescence, surface plasmon resonance, Raman scattering, and interferometry, as well as new trends in photonics are reviewed. The challenges of materials selection, surfaces tailoring in microfluidic structures, and enhancement of the sensitivity and miniaturization of biosensor systems are discussed. The review provides an overview for current advances and future trends in microfluidics integrated technologies for label-free protein biomarkers detection and discusses existing challenges and a way towards novel solutions.

## 1. Introduction

Proteins are by far the most important biological macromolecules playing a unique role in the functioning of any living being. Many processes inside the cell are bound to specific types of protein molecules. Because of their significance, the detection, quantification, and characterization of proteins are among the most common tasks in medicine and biology, both in benchtop research and in clinical practice [1]. Highly sophisticated equipment and extremely sensitive techniques [2], such as gel electrophoresis and isoelectric focusing [3,4,5,6,7], high-performance liquid chromatography (HPLC) [8,9], mass-spectrometry [10,11,12,13,14], western blotting [15,16,17], enzyme-linked immunosorbent assay (ELISA) [18,19,20], nuclear magnetic resonance (NMR) spectroscopy [21,22,23], isotope labeling [24,25,26], light scattering [27,28], chemiluminescence [29,30,31], circular dichroism [32,33,34], and optical spectroscopy [35,36,37] are widely used in proteomics research. 

The number of proteins comprising the human proteome is estimated between ten thousand and several billions, most of them are still unidentified or their functions remain unclear [1]. Multiple international projects have been realized for exploring the human proteome, but the undertaking is far from being finished [38,39,40,41]. Meanwhile, in practical medicine, only a limited number of clinically significant proteins are determined in biological fluids (blood, urine, saliva, cerebrospinal fluid) and tissues by routine clinical tests [42,43]. Many protein biomarkers could be detected (often at a single molecular level) before any clinical manifestations of a pathological condition, giving an indispensable tool for prediction and early diagnosis of tumors, metabolic and endocrine deceases, fetal abnormalities, etc. [44,45,46,47,48,49]. One of the main challenges in the field of protein biomarkers detection is the gap between research results and clinical application with an affordable, reliable, and easy-to-use techniques and equipment [50]. 

There is a growing tendency towards using portable analytical biosensors for point-of-care (POC) diagnosis, including lab-on-chip systems, as an alternative to conventional protein assays, which are used in full-scale clinical laboratories equipped with automated biochemical analyzers. POC systems are closer to patients and doctors, more affordable and convenient for small hospitals, and often present the only option in remote or sparsely populated areas. POC devices based on microfluidic technologies are on target to become the most portable, efficient, and cost-effective option; relatively small-volume samples are required for analysis with such sensors [51,52,53]. Due to their advantages, microfluidic platforms have also become popular in biological and biochemical research, drug discovery, and the synthesis of pharmaceutical substances. 

Protein biosensors often rely upon labeling procedures involving selective interaction of protein molecules with specific antibodies, which are bonded with an easily detectable enzyme molecules (ELISA), fluorescent dye or quantum dots (fluorescent labels), functionalized biointerface (acoustic sensors), etc. In historical perspective, labeling techniques (especially fluorescent labeling) allowed for the achieving of previously unattainable sensitivity and specificity and became the gold-standard in biochemistry. Yet, despite the obvious advantages, labeling is often a cumbersome, expensive, and demanding process, inevitably and inadvertently altering the structure of a protein molecule.

In some cases, higher affordability can be achieved while using analytical devices based on solely physical label-free techniques of protein detection. This approach allows for the developing of biosensors with minimal requirements for sample preparation and expensive chemical reagents. Apart from practical and economic advantages, they provide unique possibilities to detect and characterize proteins in their native state, and to investigate protein structural conformations and molecular interactions. In spite of all the benefits, label-free techniques are still less popular for POC devices applications. The challenge is the difficulty to achieve the same sensitivity and specificity compared to the labeling approaches.

This review is solely devoted to label-free techniques and methodologies based on different physical phenomena for proteins detection, quantification, and characterization in microfluidic biosensor platforms. It should be noted that methods for protein sorting and separation, e.g., electrophoresis, chromatography, acoustophoresis, deserve a separate comprehensive review and lie beyond the scope of current work.

In Section 2 of the review, the methods for manufacturing microfluidic structures for biosensors are discussed, including lithography, the oxidation of silicon, chemical vapor deposition, chemical structuring of substrate material, and 3D printing. Section 3 gives an overview of optical and spectroscopic methods and microfluidic structures for protein detection: optical properties of proteins, ultraviolet, visible, and near-infrared (UV-Vis-NIR) absorption spectroscopy, Fourier-transform infrared (FTIR) spectroscopy, fluorescence spectroscopy, surface plasmon resonance, and surface-enhanced Raman spectroscopy. Along with conventional off-chip systems, special emphasis is made on optofluidics as a perspective new trend in optical detection. Section 4 is focused on impedance spectroscopy microfluidic techniques and methods for proteins detection.

## 2. Methods for Manufacturing Microfluidic Structures for Biosensors

### 2.1. Microfluidics: Basics and Materials

Microfluidics is a direction where microsystems technology-based solutions are developed for the precise transport and manipulation of fluids (liquids or gases) in small volumes, as microliters [54]. This is the main advantage of the microfluidics approach as a field of science where current advances reach picolitres handled volumes [54]. Microfluidics has experienced a significant development over the last decades following the advances in material science, chemistry, micro and nanostructuring, etc. Due to the improvement of material properties, the discovery of new chemical methods of surface passivation [55], and the development of methods and devices for high-precision structuring [56], it becomes possible to operate with ever smaller volumes of liquids and to implement sensors, optical elements, and micromechanical manipulators into the channels. All this opens new horizons for accuracy, reproducibility, and speeds up the manipulation and analysis of biological liquids. This is especially important for working with biological fluids and detecting proteins in biomedical applications. Furthermore, the absence of dead volume minimizes consumption of valuable reagents. Together with small amounts of the required material under test (MUT), it reduces the costs of experiments as a result. 

Another advantage of microfluidic devices is the laminar flow conditions, which is characterized by low values of the Reynolds number. It allows greater flow control, has a linear relationship between flow rate and pressure, and the flow is characterized by non-crossing streamlines. The impact of the flow type is essential for e.g., surface plasmon resonance biosensing (which is described in Section 3). Also, laminar flow reduces the amount of air bubbles in the channel and noise [54].

The geometrical configurations of microfluidic platforms can be very different (for example, channels, tanks, separators, mixers, etc. [57,58]) and depend on the application. If microfluidic devices are created only for detecting certain biological components, they usually have a simple geometric structure in the form of one or more channels with sizes from micrometers to millimeters in cross-section. Such channels are provided with an inlet and outlet for liquid, which can be introduced to the channel using a pipette, a pump, capillary forces in the presence of the hydrophilic properties of the chip material, or by creating backpressure at the outlet. Also, a channel can be equipped with different additional components due to the sensing mechanism, e.g., lenses, micromanipulators, electrodes, etc. 

In some applications, the task is not only to detect, but also to manipulate the biological sample. For example, it is necessary to sort out certain components or particles to increase the concentration of the detected component. In this case, the geometry of the microfluidic system will be more complex and passive, or active separation structures can be added to the chip [57,58]. For labelled techniques, the microfluidic chip can be expanded with microfluidic mixers [59] to blend the material under test (MUT) with fluorescent components or nanoparticles. In this review, we focus on label-free detection of proteins in biological fluids, so we will not delve into the components for manipulation. Here we look at microfluidics as a platform for holding small volumes of liquids, its microstructuring, and sub-micron structuring techniques. We also turn to the material properties of a microfluidic chip, which are necessary for optical and dielectric methods of detecting proteins.

The most important common properties, in this case, are biocompatibility and low protein adsorption. Biocompatibility is considered here at the level of inertia of the structure materials towards biofluids in physical contact. Based on the same considerations, the following property is mandatory—the absence of proteins adsorption.

For any application the microfluidic chip materials have to be chosen carefully for their applicability in detecting proteins using the methods described below, the complexity and costs of structuring, biocompatibility, and the lack of absorption of the test material. Therefore, for the optical label-free protein detection is important that chip-body will be transparent in UV, visible and infrared (IR) spectrums, especially at the wavelength range 250–350 nm and middle IR region 2.5–20 µm. Several glass types, such as quartz glass, for example, are widely used in microfluidics with optical sensing in the UV spectrum. Borosilicate glass is also transparent in UV and at the same time in deep IR range up to the 2.9 µm wavelength. Vycor^®^ glass at the thickness of 1 mm is transparent up to 3.1 µm. Furthermore, glass has a very low protein adsorption in comparison to polymers. At the same time, polymers are transparent at the VIS spectrum, but they are inferior to glass in UV and IR transmittance characteristics [60]. The main advantage of polymer materials for microfluidics is their relatively easy structuring. Thus, the production costs are much lower compared to glass structures. Precisely because of the simplicity and cheapness of structuring, polymers are the prevailing material of microfluidic structures with dielectric and electrochemical impedance based biosensors. Along with it, often are used material combinations, e.g., polymer/glass or polymer/silicon.

There are several examples of the sensor-microfluidic chip using a combination of the aforementioned materials. To embed the sensor into the microfluidic structure, a hard substrate is typically used as a sensor carrier. The µ-structured silicone or plastic layer is bonded on top of the sensor embedded in the carrier. Hu et al., fabricated and combined a microfluidic chip with a micro-spectrometer. The authors report the preparation of the optical system by a combination of e-beam lithography and dry etching pattern transfer processes. The microfluidic Polydimethylsiloxane (PDMS) channel was structured with molding technology, aligned, and bonded on a prepared wafer using a structured optical system [61]. Horrer et al., report about the plasmonic optical biosensor integrated into a micro-fluidic cell. Also, in this work, the optical structure is made of glass and the microfluidic channel is realized in PDMS [62]. Alsabbagh et al, reported an impedance-based biosensor realized on a glass substrate bonded with a PDMS microfluidic channel [63]. Bavil et al., used the same material combination to realize a microfluidic microparticle-labeled impedance sensor array for enhancing immunoassay sensitivity [64].

As one can see, the most common polymers for microfluidic devices are polydimethylsiloxane (PDMS), polyester, polycarbonate, etc. [65,66]. The reason for this is that silicones and plastics are non-toxic, transparent, and easy to structure. The most outstanding material is the PDMS, since, in addition to the aforementioned properties, it is gas permeable, flexible, and it can be structured using soft lithography prepared molds (see Section 2.2.1 and Section 2.2.7). As opposed to that, the alternative solutions using the aforementioned mm-thick layers of plastics (polyester, polycarbonate, etc.) have to be structured by hot embossing technology, which requires a costly stamp for the desired geometry. Hot embossing is a method which is based on impressing the plastic by a prepared metal template under certain temperature and pressure conditions. It is also possible to apply UV light or ultrasound vibrations instead of heat, in combination with mechanical force in cases where it will improve the manner of material structuring.

An important challenge that has to be addressed is the enabling of the integration of the sensors into microfluidic platforms and readout electronics. There are several sensor approaches to detect proteins in situ in microfluidics by means of optical or dielectric and electrochemical impedance spectroscopy [67]. Realization of readout electronics for these sensors requires highly-integrated miniaturized circuits realized in advanced semi-conductor technologies (e.g., complementary metal-oxide semiconductor (CMOS) technology). This would contribute to enhancing the reliability of sensor reading and enable controlling the sensors externally or wirelessly. Additionally, CMOS readout electronics would provide an advantage in terms of providing new opportunities for packaging realization. This is due to the fact that miniaturization of the control and readout electronics reduces the size of the module and enables more compact and feasible packaging solutions of the entire module, including a biocompatible microfluidic chip and integrated sensor. Impedance characterization of electrodes can be done by the classical method presented in [68]. Sensors embedded into microfluidics requires de-embedding of the interconnects between the measurement equipment (e.g., vector network analyzer (VNA)) and the terminals of the sensor. Advanced de-embedding techniques can be applied in this case [69]. By means of a miniaturized module and automated readout, one can get rid of bulky equipment by a fully autonomous system operation and wireless data transfer. The possibility to transfer the readout data wirelessly would make the system portable, and it could be even placed in an incubator, which is essential for the measurement of specific proteins, due to the requirement of regulated temperature. Some examples of an autonomous implantable integrated CMOS chip are given in [70]. Examples of building blocks required for building a wireless data communication link can be found in [71,72,73].

### 2.2. Technological Approaches for Microfluidics 

The development and production of microfluidic systems is based on the manufacturing processes of semiconductor and microelectromechanical systems (MEMS) technology. Various technical processes are used which can be both additive and subtractive in nature. In addition to lithography as an interconnection technology, fundamental processes such as wet and dry chemical etching, the physical deposition of metals or the galvanic reinforcement of metallic structures can also be found in the following section [74,75].

#### 2.2.1. Lithography as a Link between the Other Processes

**Figure 1:**
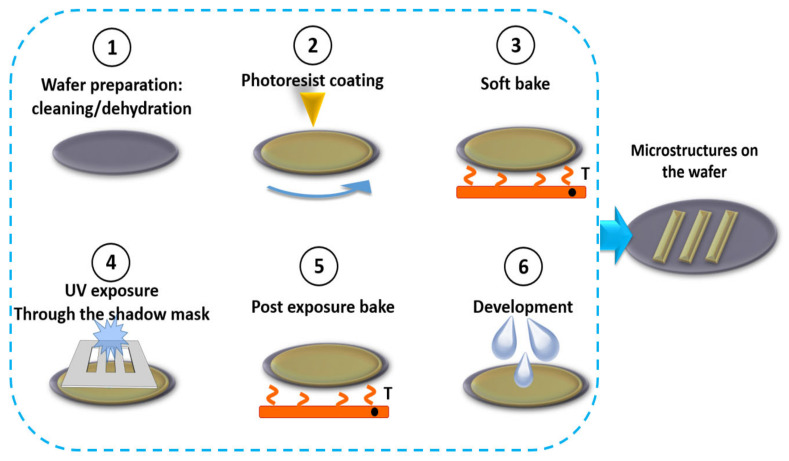
Schematic view on soft lithography process and description of the technological steps, which are wafer preparation, photoresist coating, soft bake, UV exposure through the shadow mask, post exposure bake and development.

UV lithography is an essential process for the fabrication of microfluidic components. It refers to the UV light exposure of the spin-coated layer of the photoresist through the shadow-mask. Once the layer is exposed, non-crosslinked parts of the photoresist are washed out during the development and only the crosslinked structures stay on the wafer. All the technological steps of the lithography process are shown schematically in Figure 1. It offers the possibility of transferring the previously developed design, which has often been optimized through complex Computational Fluid Dynamics (CFD) simulations, to the basic substrates. As the name suggests, this describes a process for fabricating structures with the aid of UV light. The UV light can be generated by a cross-silver vapor lamp or by aUV Light Emitting Diode (LED). Conventionally, the wavelengths of the generated radiation are in the range of 365–405 nm and are used to irradiate a photosensitive material. There are two major types of photosensitive material, positive and negative coatings. With positive coatings, the area to be exposed becomes more soluble than the non-exposed area due to the UV radiation, and can be dissolved more quickly later during the development process. The opposite process exists with negative coatings. If a negative coating, e.g., photoresist SU-8, is irradiated with UV light, a chemical reaction occurs that causes the molecular chains to lengthen and thus reduces solubility. Depending on the type of coating, these occur in different configurations, but almost all coating systems contain solvents, resins, photosensitive components and agents to optimize adhesion [74,75,76].

The actual lithography process takes place in several steps, as it is shown in Figure 1. First, the substrate surface is cleaned and freed from water. This is done in multi-stage baths (standard-clean 1 (SC1), hydrogen fluoride (HF) dip, standard-clean 2 (SC2)) to remove grease and metallic impurities. Finally, the wafers are dried on a hotplate or in an oven above 140 °C. In the second step, the substrate is usually given an adhesion promoter that is optimized for the respective photoresist and applied either by spin or steam coating. In both cases, the coating is followed by a further bake-out step to optimize the adhesion of the adhesion promoter. In the third step of UV lithography, the photoresist is applied by spin coating or spray coating. In both processes the aim is to achieve a homogeneous distribution of the coating on the substrates. The homogeneity is significantly higher with spin coating and is approx. ± 1 nm, but only permits a constant coating thickness on unstructured substrates. With spray coating, the homogeneity is usually approx. ± 250 nm, but allows coating on already structured substrates. In the so-called softbake in a convection oven or on a hotplate, the residual solvent is expelled at approx. 90–120 °C and the coating is further solidified. In the fourth step, the design structure is transferred with the help of UV light. The discussed structure area is irradiated in a mask aligner and a UV-transmitting quartz-chrome mask. The highest resolution is achieved in hard contact, where the wafer and mask are pressed together, and the poorest in the proximity method. However, the direct contact between mask and photoresist can lead to contamination or wear of the mask. As a result of the proximity method, the resolving power is reduced, but the mask and also the resist layer are protected. The distance here is between 10–20 µm. After exposure, the negative coating undergoes another heating step to cross-link the polymer chain. In the fifth step, the material to be removed is dissolved out with a lye solution or solvent and developed. In the last step, the sixth, the resist is additionally solidified once again in an oven or hotplate process to optimize adhesion or, additionally, to solidify the organic resist structure [74,75].

#### 2.2.2. Oxidation of Silicon

For the development of microfluidic structures in silicon, the oxidation of the base material is an indispensable technology. In horizontal or vertical furnaces, oxygen or oxygen moistened with water flows around the pre-cleaned silicon, dry and moist oxidation. There is almost atmospheric pressure in the furnace and the temperature is between 900–1200 °C, depending on the process application. The growth rate of the silicon, which grows 56% inwards and 44% outwards, is not constant and increases with increasing oxide thickness. This is due to the increasingly longer diffusion time of the oxygen atoms through the already existing oxide layer. Dry oxides with high layer quality are mainly used for high-quality transistor dielectrics. If, on the other hand, masking layers for etching processes are of interest, moist oxides with between 100–300 nm are often used [74,77].

#### 2.2.3. Chemical Vapour Deposition (CVD)

In addition to the oxidation of silicon, the deposition of silicon nitride, polysilicon or silicon carbide on the basic microfluidic substrates is also of interest for many applications, sensors, and protective and passivation coatings. These coatings can be deposited in several ways, with and without plasma assistance and at subatmospheric or atmospheric pressure. Chemical vapor deposition, CVD, is used for this purpose.

CVD is another basic technology and uses the gaseous reaction starting materials SiH_4_/NH_3_, SiH_2_Cl_2_/NH_3_ or SiH_4_/CH_4_, which are made to react by thermal energy or plasma support. The reaction end product is usually a solid as well as a volatile component. The solid serves as a useful layer for the applications described, and can make the substrate materials such as silicon, glasses or even polymer layers more resistant or, for example, change the wetting properties [74,78]. 

#### 2.2.4. Wet and Dry Chemical Structuring of Substrate Material

The structuring of silicon and glass is a basic technology for the production of microfluidic systems. In addition to liquid etching on an acid or alkali basis, various etching gases are also used which are specially adapted to the substrate material. 

In the 1980s, the wet chemical structuring of silicon was a fundamental technology for the development of microfluidic systems. For example, even then silicon or glass was patterned and used in printheads by Hewlett Packard [79]; silicon can be directionally patterned using potassium hydroxide (KOH). The typical pyramid shape is formed, for example, by silicon (100), and enables the production of nozzles when a substrate is completely etched through. Silicon can be structured very stably and with high reproducibility [75]. 

In addition to the wet-chemical structuring of silicon, the development of the so-called Bosch process, today also known as deep reactive ion etching, was another milestone in the further development of plasma patterning methods. In this process, the silicon substrate surface is initially covered with a photoresist mask or silicon oxide and the desired design, and then patterned using a reactive etching gas (SF_6_) in a combination of physical and chemical attack. In the second etch process subsection, complete passivation of the substrate surface is performed using a plasma-generated polymer of C_4_F_8_ and argon (carrier gas). The generated polymer layer covers both the etch trench bottom and the existing etch trench wall. If the combination of physical and chemical etch attack is now performed, the etch trench bottom and the etch trench wall are etched more intensively. Thus, it is possible to etch nearly vertical structures into silicon with a defined wall roughness and given structure at width to depth ratios of 1:100 and more [74,78,80].

#### 2.2.5. 3D Printing of Microfluidic Systems

The technological development and realization of microfluidic systems is not limited to the common semiconductor and MEMS processes but is also possible with a wide variety of 3D printing processes. The associated processes use, among other things, curing resists or remelting polymers. The following is a brief description of the most important processes for manufacturing microfluidic systems in 3D printing [81,82,83,84].

##### FDM or FFF Printing

The pioneer in the development of fused filament fabrication or fused deposition modeling (FFF or FDM) systems was the company Stratasys. This process was developed in 1988 by S. Scott Crump, a co-founder of Stratasys. As the name suggests, the object to be produced is made from a fused wire. The wire, usually a plastic, is melted and pressed through a nozzle. This nozzle can be a few hundred µm or even a few millimetres in diameter and is movably suspended above a heated build plate made of glass or metal. When the filament, which has been heated above its liquefaction point, hits the heated construction plate, it solidifies and allows the partially cooled polymer to be built up layer by layer. The structural accuracy of the manufactured object image depends on several parameters, including nozzle diameter, layer height, processing temperature of the filament, build temperature, build plate temperature, etc. [84].

The materials used depend directly on the intended use and the technical possibilities of the printer. Thus, it is possible to use low-melting and at the same time very inexpensive polylactic acid (PLA) for rigid and simple structures without great technical demands. Even with low-priced printers, it is possible to process PLA [85]. If the technical requirements increase due to lower water absorption and temperature resistance, acrylonitrile butadiene styrene (ABS), or polycarbonate (PC), are used. However, the processing demands increase, for example, in the pressure temperature from 200 °C to 230 °C or 270 °C for PC [86]. The processing of high-performance polymers is also possible today. For example, production of microfluidic elements in polyetheretherketone (PEEK) can be realized at 440 °C. Printed elements made of PEEK also enable the analysis of corrosive media [87,88].

One obstacle of FDM technology is the production of overhangs. In many cases, this is only possible with the help of support material. This support material temporarily stabilizes free-standing areas of the print during the printing process and sometimes considerably extends the processing time. After printing, this support material must be removed in an additional post-treatment step; this can be done mechanically (breaking, grinding) or chemically (dissolving with water or lye solution). An environmentally friendly and at the same time water-soluble support material is polyvinyl alcohol (PVA) [89]. Unfortunately, FDM 3D printing has several disadvantages such as comparably low printing quality and speed, and layer-by-layer FDM printing can lead to shrinking and warping.

As already mentioned, the minimum print resolution depends on many different parameters (design, capabilities of the printer, print material). For example, the structure size is <0.1 mm and the layer height of <0.1 mm and wall thicknesses of <0.1 mm [90].

##### Stereolithography (SLA) Printing

The stereolithography process, or SLA process for short, is another method for creating microfluidic structures. Here, however, the polymer to be used later is not thermally melted but cured with the help of light quanta. The polymer mixtures to be used (resin) have a photoactive component and can be cured with the help of a laser. The printing plate is usually immersed upside down in the polymer. The laser then writes the first support layer on the plate surface to improve adhesion between the resin and the base plates. After completion of the first adhesion layer, the solidified polymer layer is pulled out of the liquid polymer matrix by a few µm, and the next structure is cured with the laser on the already solidified surface. The step-by-step production, i.e., layer by layer, is similar to the FDM process. Here, too, self-supporting structures are only possible with the help of support structures. However, in contrast to FDM, no second water-soluble material can be used for SLA printing. After printing, excess resin residues are washed out with IPA, isopropanol, and the support structures are removed with mechanical aids [83,91].

The structure resolution of approx. 25 µm is significantly lower than that of the FDM process.

#### 2.2.6. Sub-Micron Structuring

**Figure 2:**
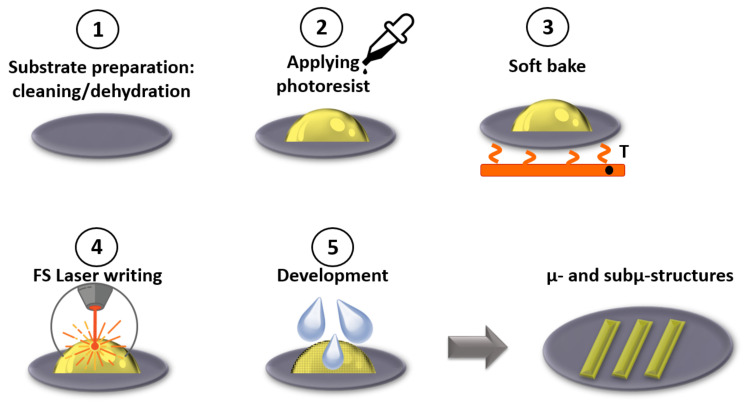
Schematics of 3D laser lithography 3DLL technology, which is based on next steps: substrate preparation, applying photoresist by pipet, soft bake on the hot plate, femto-second laser writing, and development in the liquid developer. The development step (5) reveals the final free-standing micrometer or sub-micrometer size structure.

Recent developments in lasers opened up a new era for mesoscale microfluidics. Two-photon polymerization based on 3D laser lithography (3DLL) applied to micro-optics [92], microfluidics [93] and photonics [94] allow for the achievement of nano- and micro-feature enabled functionality in micro-structures. 3DLL is a flexible manufacturing method, and it is possible to print almost any kind of sub-micron 3D object with nano-resolution. As it is a laser lithography process, the structured materials are polymers. Nevertheless, a wide range of materials allows finding the optimal one for the chosen application. Using this method, it is possible to structure the 3D objects even inside of the fabricated microfluidic channel [95]. A schematic view of the 3DLL technology is shown in Figure 2.

Glass is a more appropriate material for optofluidics. Laser-structuring is applicable in this case as well, but it works differently than lithography. It “grinds” an object of the desired shape from a piece of glass or a special light-sensitive material, and does not expose it layer by layer. Such an approach was applied to fabricate micro-lenses with submicron precision. To make a lens it is necessary to apply several layers of special material to the glass disc which will prevent the evaporating of the glass or polymer, and then process it with femtosecond pulses, reapply the “protective layer” and repeat this procedure until the lens is ready.

As a demonstration, we created a miniature (1.4 by 1.4 micrometers) camera containing 1600 pixels by printing lenses directly onto the surface of the microchip and lenses 120 micrometers in diameter and 100 micrometers thick [96]. Furthermore, in 2018 we reported the deterministic integration of a preselected single InAs quantum dot (QD) into an on-chip 50/50 multimode interference (MMI) beamsplitter via in-situ electron beam lithography (EBL) [97].

#### 2.2.7. Molding and Bonding 

Molding technology is used for polymer structuring. Polydimethylsiloxane (PDMS) is one of the most well-known materials which can be structured by this method. It is highly hydrophobic, is transparent in the visible spectrum, is flexible and biocompatible. These characteristics made PDMS attractive for several biomedical applications and microfluidics [98]. 

**Figure 3:**
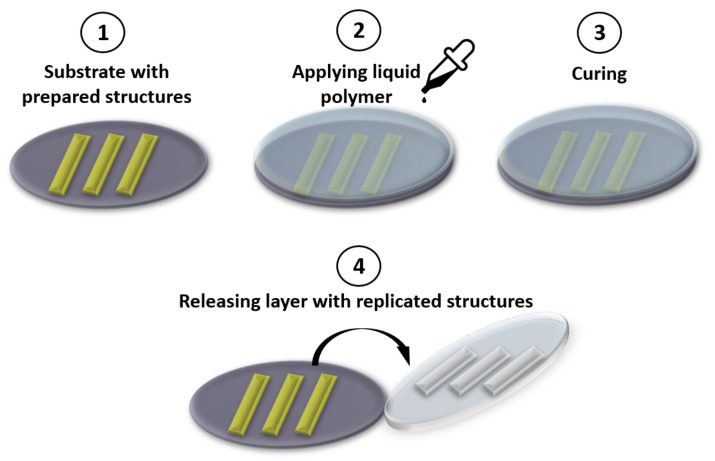
Schematics of the molding technology. The liquid polymer is applied on the prepared mold (2), cured (3) and released (4) with the replicated from the mold structures.

Molding technology consists of the following steps: (1) Polymer preparation (liquid phase); (2) Pouring out or spin-coating liquid material on the master mold; (3) Curing; and (4) Releasing the structured layer, which replicates the master mold geometry (Figure 3). In the PDMS example, as a first step, the base and curing component have to be mixed in a ratio of 10:1. Viscous material has to be poured out on the preparation by soft lithography mold. After 48 h of curing at room temperature or 2 h at 60 °C, the structured layer can be released. Next, half of the microfluidic channel has to be bonded to any structured or non-structured layer.

Bonding is a technological process intended to combine several layers of carrier material. Upon completion of the bonding process, these layers are merged into a single unified microfluidic structure. The surface of the two layers, which need to be bonded, is usually exposed to a plasma or chemical treatment, adhesives, and temperature treatment. It creates active groups on the material surface which form a covalent connection to another layer. One can specify these steps for bonding of the PDMS and glass. This type of polymer requires the oxygen plasma treatment, then alignment of the two layers and heating up to 80 °C for 10 min. We describe this configuration as it is quite common for microfluidics with embedded impedance sensors [99]. The sensor electrodes are deposited on the glass surface and the microfluidic channel is bonded on top. The disadvantage of PDMS is protein adsorption due to its loose nature. During the analysis of the liquids with high protein concentration it does not contribute to the result value. But for the precise measurements, another transparent polymer can be chosen that is less elastic and adsorbing. For the hard polymers, structuring can be applied by the hot embossing method and adhesives to provide reliable bonding. For some of the photoresists, UV exposure and heat/pressure treatment are needed to achieve a covalent bond [100].

#### 2.2.8. Surface Functionalization to Avoid Protein Adsorption

The ability of the materials to adsorb proteins in the thin layer below the surface de-pends on their structural properties. Glass exhibits properties of the crystalline structure, therefore, the protein adsorption is very low, and it is used for many biological processes and applications. Polymers have a structure of polymer chains which can be differently orientated and entangled. Also, polymer chain density and porosity can vary strongly from one polymer to another. Therefore, polymers are not the ideal material for biological applications, as they adsorb and absorb a wide variety of molecules. 

The previously mentioned polydimethylsiloxane (PDMS) is a porous and hydrophobic polymer material [101]. PDMS’ porosity is a result of the flexibility of its polymer chains and the large distance between them. Thus, it has a high level of protein adsorption and absorption [102]. Due to the variety of beneficial properties mentioned above, PDMS is a commonly used material in micro-fluidics. Therefore, several interesting approaches to modify its surface were developed. Recently, Gökaltun et al., reported an interesting method to avoid protein adsorption in PDMS. The authors added an additional component during the mixing of PDMS base with a curing agent. As a result, the surface becomes hydrophilic only in the presence of liquid in the channel, and self-assembling smart copolymers comprised of poly(ethylene glycol) (PEG) block the adsorption of the particles [55]. Ren et al., created an approach to avoid salinization of the PDMS and achieve low protein adsorption and hydrophilicity. They fabricated wax-modified PDMS channels with the paraffin peristaltic valves for micellar electrokinetic chromatography. The alternative methods to prevent adsorption and absorption of the proteins are Teflon coating of the surface, water-repellent spraying, and perfluorodecyl trichlorosilane (FDTS) blending [103]. You et al., developed a modern method to avoid the absorption of fluorescent molecules into PDMS. Therefore, we created a nanoadhesive layer deposited via initiated chemical vapor deposition (iCVD) [104]. We can conclude that there are several ways to modify the polymer surface to prevent protein adsorption and absorption. All of them are based on adding chemical components to cover the surface or to be added into a polymer.

## 3. Label-Free Optical Techniques of Protein Detection, Quantification, and Characterization in Microfluidics 

Optical detection, quantification, and characterization techniques dominate in microfluidics, including a novel and rapidly evolving area of lab-on-chip biosensing platforms for biological and medical applications, due to a number of fundamental and practical reasons, such as non-invasiveness, versatility, high sensitivity and specificity, robustness, relative affordability of analytical equipment, easy coupling of optical elements and microfluidic structures. Numerous books and journal reviews have been published on this topic in the recent years and the interest is constantly growing [105,106,107,108]. Protein research is by no means an exclusion from this trend; it has been widely demonstrated that reliable and well-established optical and spectroscopic methods, which are extensively used in analytical biochemistry for protein determination and characterization, could be successfully implemented in microfluidic platforms [51,52,53,54]. Various simple and more sophisticated techniques such as UV-Vis-NIR spectrophotometry, Fourier transform infrared spectroscopy (FTIR), fluorescence spectroscopy, light scattering, chemiluminescence, refractometry, reflectometry, surface plasmon resonance (SPR), and surface enhanced Raman spectroscopy (SERS) could be used in microfluidics depending on the required limit of detection (LOD), signal-to-noise ratio, interference from other substances, etc. [109,110].

Numerous original research articles and excellent reviews highlight achievements in the field of optical biosensors for protein detection. Some papers consider common trends in analytical methods and biosensing technologies, i.e., in the work [111] (2021) main strategies for further increasing sensitivity of protein determination are overviewed. The comprehensive review [112] (2020) presents novel approaches for optical biomolecules characterization, including proteins and nuclear acids, the paper [113] (2020) is devoted to rapidly growing smartphone-based biosensor applications, the reviews [114] (2020) is dedicated to optical sensors for biomedical diagnostics in general (including proteins), the work [115] (2017) is specifically focused on emerging applications of label-free biosensors in protein and DNA research. 

The other type of reviews are concentrated on a particular sensing technology or active substrate, i.e., photonic crystals-based biosensors for biomarker detection [116] (2021), biosensors with graphene substrates [117] (2021), [118] (2019), fluorescent sensors for single-molecule protein detection [119] (2021), [120] (2018), aptamer-based biosensors [121] (2020), [122] (2019), array-based discriminative optical biosensors for identifying multiple proteins in biofluids [123] (2020), sensors based on whispering-gallery mode (WGM) microresonators [124] (2020), label-free plasmonic [125] (2020), [126] (2019) and interferometric [127] (2019) biosensors, platforms for lateral flow quantitative assays [128] (2019), novel SERS substrates [129] (2018), label-free optical resonant sensors for biochemical applications [130] (2013), quantum dots-based biosensors [131] (2018), and colorimetric sensors for rapid detection of protein [132] (2018). Some reviews are dedicated to optical components for biosensors: micro-optics elements for microfluidic analytical applications [133] (2018) or microfiber-based microfibre based photonic components and their applications in label-free biosensing [134] (2015).

Many works have a narrower scope and overview the variety of analytical methods and biosensing technologies for detecting specific biomarkers (proteins, peptides, enzymes) or pathogens: cancer biomarkers [135] (2021), [136] (2021), [137] (2021), [138] (2018), rheumatoid arthritis (Ra) biomarkers [139] (2020), Alzheimer’s biomarkers (tau-protein) [140] (2020), [141] (2020), inflammation markers, (C-reactive protein) [142] (2020), [143] (2020), [144] (2018). 

It should be noted that among a plethora of publications mentioned above only a relatively small share is specially dedicated to label-free optical analytical techniques, biosensing technologies and design. In our opinion comprehensive reviews [114,116,122,126] deserve a special attention.

Unlike many small molecular substances, a majority of proteins manifest quite moderate and non-type-specific optical activity in the UV-Vis-NIR region, which is the most convenient for optical detection from the point of view of instrument design; the origin of this phenomenon will be briefly discussed later in this review (Section 3.1). As a result, characteristic electron absorption, intrinsic fluorescence, or Raman scattering of proteins (particularly in solutions) are relatively weak and often cannot be associated with a particular type of protein in mixtures or complex media. This is the main factor limiting sensitivity and specificity of direct (label-free) optical methods for protein detection, especially in microfluidic structures with extremely low analytical volumes. Different methodological approaches are used to overcome this problem by enhancing optical signals and providing specific response from a particular protein:

- labeling-binding (constantly or temporarily) to a protein molecule a highly optically active (most often in the visible region) foreign molecule, quantum dot or molecular complex, e.g., by specific antibody-antigen chemical interaction [145];
- molecular sorting of different proteins in a mixture (using separation or focusing) on an analytical stage prior to detection, e.g., by electrophoresis, isoelectric focusing, acoustic waves, or chromatography;
- manyfold amplification of an optical signals emploing surface plasmon resonance, surface enhanced Raman spectroscopy, and surface-enhanced infrared absorption spectroscopy.

Generally, label-free (direct) techniques have higher LOD, and worse specificity compared to labelling methods, but preserve the molecular structure intact, which gives them the unique possibility to detect and characterize proteins in their native state and to, investigate protein structural conformations and intermolecular interactions [146,147]. This chapter of the review is solely devoted to label-free optical techniques with the emphasis on microfluidic platforms for protein research. 

In Section 3.1, optical properties of proteins in the UV, visible and IR regions, which impose fundamental limitations on sensitivity and specificity of optical detection methods and to a great extent define their advantages and drawbacks, are briefly analyzed. Descriptions of various well-established label-free methods are presented in the order towards increasing sensitivity and specificity: UV absorption spectroscopy, MIR spectroscopy, intrinsic fluorescence, refractometry, surface plasmonic resonance and surface enhanced Raman spectroscopy. 

Section 3.7 is dedicated to diffusometric methods for protein characterization, which are important in protein research, but not so easily implemented in micro-fluidic structures as the techniques mentioned above. Section 3.8 is devoted to optofluidics sensors for protein detection as an emerging technology.

### 3.1. Optical Properties of Proteins in the UV, Visible and IR Regions

Attenuation of optical radiation propagating in a medium is a result of two fundamental physical phenomena of light-matter interaction—absorption and scattering; according to the Beer-Lambert law the intensity of radiation *I*_λ_ decreases exponentially [148]:

(1) $$I_{\lambda }=I_{0\lambda }\exp\left[-\mu_{e}\cdot d\right]=I_{0\lambda }\exp\left[-(\mu_{a}+\mu_{s})\cdot d\right],$$

where *I*_0λ_ is the intensity of incident radiation; µ_a_—the bulk absorption coefficient; µ_s_ is the bulk scattering coefficient; µ_e_ = µ_a_ + µ_s_ is the extinction coefficient, which includes both absorption and scattering; *d* is the thickness of an optical layer. The bulk absorption coefficient of a solution is directly proportional to the concentration of a solute $\mu_{a}=\varepsilon_{\lambda }C$, which in the case of a low turbidity medium can be found from the sample transmittance $T_{\lambda }=\frac{I_{\lambda }}{I_{0\lambda }}\cdot 100\%$:

(2) $$C=\frac{1}{\varepsilon_{\lambda }d}ln\left(\frac{I_{0\lambda }}{I_{\lambda }}\right)=\frac{1}{\varepsilon d}ln\left(\frac{100\%}{T_{\lambda }}\right),$$

where *ε_λ_* is a molar extinction coefficient.

The overwhelming majority of proteins, including the most abundant in biological fluids, globular plasma proteins (albumin and immunoglobulins), are colourless in water solutions, virtually transparent in the visible region and show very weak absorption in the near infrared range (NIR). Some conjugated proteins, e.g., haemoglobin, are an exception from this rule, but their protein carriers are also colourless [149].

Proteins actively absorb optical radiation in the UV region with two characteristic peaks around the wavelengths of 280 nm and 200 nm [150]. Absorption in the longest wavelength band 250–300 nm mostly arises from aromatic amino acid residues—tryptophane, tyrosine, phenylalanine—and to a much lesser degree from the disulphide bond in cysteine. There is a high level of variability of absorbance at 280 nm from protein to protein because of the different content of aromatic amino acids and structural differences in protein macromolecules; some proteins with low tryptophane content show very little absorption at the wavelengths higher than 250 nm [151]. The peptide bond is responsible for the short wavelength absorption below 210 nm; generally, in this area the variability between different proteins is much less prominent and the absolute absorbance level is significantly higher than at 280 nm [152]. 

Ultraviolet absorption of various proteins was intensively investigated in the 1950s, 1960s and 1970s; very substantial reviews summarizing multiple original papers in this area were published by D. Kirschenbaum. To give a basic overview, specific absorption coefficients of several of the most abundant or otherwise biologically important proteins are summarized in Table 1.

**Figure 4:**
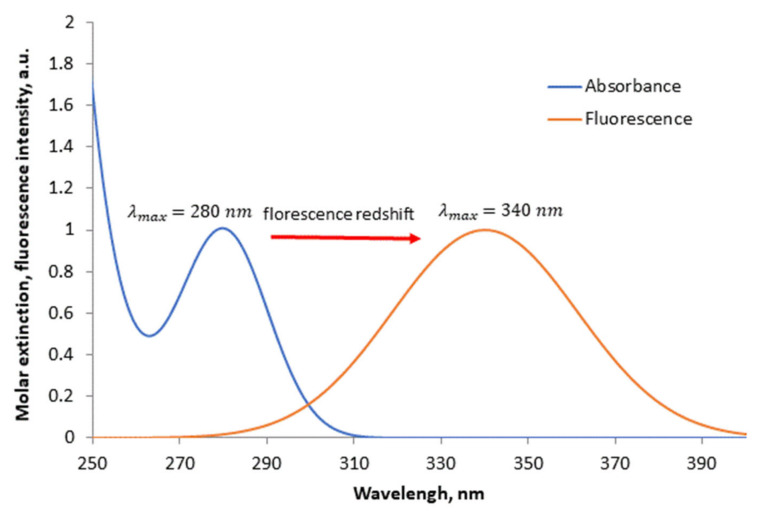
Absorption and intrinsic fluorescence spectra of HSA in water solution (a.u.—arbitrary unit).

Protein intrinsic fluorescence (autofluorescence) in the UV region is also originating from aromatic amino acid residues: tryptophane, tyrosine, and phenylalanine; optical properties of these amino acids are presented in Table 2. The contribution of phenylalanine is negligibly small due to its low absorption coefficient and low quantum yield of fluorescence; both tyrosine and tryptophane absorb around 280 nm, which is the most common excitation wavelength for protein autofluorescence [160]. Despite comparable quantum yields, the tryptophane signal is dominant because of tyrosine weaker absorption and higher internal quenching. The redshift for tryptophane fluorescence is highly dependent on a particular protein structure and surrounding, the maximum emission wavelength can vary from 303 to 350 nm, usually being near the wavelength 330–340 nm [161]. For example, the absorption and fluorescence spectra of human serum albumin (HSA) are almost solely defined by tryptophane emission (Figure 4).

It should be noted that in contrast to absorption spectroscopy, fluorescence measurements are almost always multidimensional. The parameters that are registered in fluorescence spectroscopy include excitation spectra, fluorescence spectra, quantum yield, redshift, lifetime, quenching etc. Potentially, fluorescence spectroscopy can give more information about the analyte and it is more sensitive to the protein structure and surroundings than UV spectrophotometry.

In the middle infrared region (MIR) from 2.5 μm to 20 μm proteins reveal multiple characteristic fingerprint vibrational bands associated with amino acids chains and peptide linkage (amide bond) absorption [162]. The strongest and most significant in IR protein spectroscopy MIR bands are listed in Table 3; amide bond vibrations are native to all proteins and give information about secondary conformation and solvation. The amide I band is the most useful because its line shape reflects the different types of secondary structure elements and it is not strongly influenced by side chains. An infrared spectrum provides a wealth of information on protein macromolecules: absorption properties are different for various protein secondary structure elements (e.g., α-helices, β-sheets, random coils, and loops). MIR spectroscopy is an indispensable tool for the experimental research of protein structure, conformations, and interactions [163,164]. Some of the IR techniques, e.g., FTIR ATR, time-resolved IR spectroscopy, or more sophisticated plasmonics based surface enhanced IR spectroscopy are suitable for integration in microfluidic platforms [165].

The structure of protein vibrational bands is also accessible via Raman scattering spectra. Raman spectroscopy provides unique signatures for various secondary and tertiary structures like helices, beta-sheets, turns, random structures, etc., and various amino acid residues such as tyrosine, tryptophan, and phenylalanine [166,167]. Moreover, proteins having different structures can be distinguished using specific Raman signatures without labelling [168]. Unfortunately, optical signals from protein molecules in classical Raman spectroscopy are extremely weak and internal amplification, e.g., by SERS, are needed to enhance sensitivity in microfluidic applications [169]. 

### 3.2. Overview of the Optical Techniques and Microfluidic Structures Design Strategies for Label-Free Protein Detection

There are two main strategies in the development of microfluidic chips with optical detection: free space (off-chip) systems and integrated (on-chip) structures [105,106,107,108,109,110]. In off-chip devices, optical and optoelectronic units (light sources, optical fibers, lenses, mirrors, photodetectors, spectrometers etc.) are completely separated from a microfluidic structure; optical radiation propagates in a free space before and after interaction with an analyte in a microfluidic channel. Implementation of optical detection methods in off-chip sensors is quite similar to classical analytical instruments with the exception of extremely small detection volumes compared to standard optical cuvettes [105]. Off-chip sensors provide greater flexibility in the choice of a microfluidic chip substrate and optical elements; the chip can be fabricated from both optically transparent and non-transparent materials, but in the latter case it requires transparent optical windows which are integrated into the wall of a microfluidic channel.

On-chip strategy implicates integration of microfluidic and optical elements in a single chip. It can be done either on a common substrate (optical units surrounds microfluidic channels) or in a set of separate layers; if optical components are merged with or made of fluids such systems are called “optofluidic”. This approach is much more novel and promising, it exploits the advantages of microfluidics and integrated optics at the same time: much greater degree of miniaturization compared to off-chip systems can be achieved, and complicated operations of coupling and aligning of optical and microfluidic components are eliminated from a fabrication process [170].

In subsequent sections of this chapter, applications of various optical techniques for label-free protein detection, quantification and characterization in microfluidic devices are reviewed; more attention is paid to traditional off-chip platforms. Some aspects of on-chip systems are discussed in Section 3.8. 

### 3.3. Protein Detection by Absorption Spectroscopy

#### 3.3.1. UV Absorption Spectroscopy

Methods for protein detection and quantification by measuring intrinsic UV spectral absorption (UV spectrophotometry) have been established decades ago and are still extensively used in analytical biochemistry. Most conventional protein assays employ either the single wavelength 280 nm or various combinations of two wavelengths, e.g., 280/235 nm [171] or 280/260 nm [172], which belong to the aromatic amino acids’ absorption band in protein spectra. Despite the fact that the peptide bond absorption at 205 nm is almost 60 folds stronger and gives much greater sensitivity and lesser variability between different types of proteins, it is extremely prone to interference from multiple residual substances, especially in samples extracted from native biological fluids and, as a consequence, suitable only for highly purified samples [155,173]. 

Label-free UV absorption spectroscopy is not specific to a particular type of protein. Due to the nature of protein absorption in the UV region, it is not possible to discriminate signals from different proteins in complex solutions: the method is not suitable for unprepared protein mixtures and requires additional sorting and separation procedures on the previous analytical stage, e.g., by capillary electrophoresis [174], free flow electrophoresis [175] or chromatography [176]. 

Microfluidic chips with absorption detection are usually coupled with optical units via optical fibers with collimating microlenses; additional UV transparent windows made of quartz or other suitable material are often required if the chip substrate material is UV- blocking [177]. There are systems that detect only single wavelength absorption with a monochromatic UV light source (filtered mercury lamp radiation or deep UV LEDs) and a simple photodetector; they are the most efficient, technically simple, and affordable for quantitative protein determination at 280 nm. More advanced sensors use spectroscopic detection with a wide spectral range deuterium lamp and a fiberoptic compact spectrometer, which can give more information about the shifts of absorption peaks depending on the surrounding medium, residual peaks from other components of a sample, etc. Along with conventional micro-spectrometers based on diffraction gratings, more compact and efficient systems with MEMS controlled Fabry-Perot scanning interferometers and interferential filters micro-arrays are actively implemented for spectroscopic detection in microfluidic devices [178,179,180].

**Figure 5:**
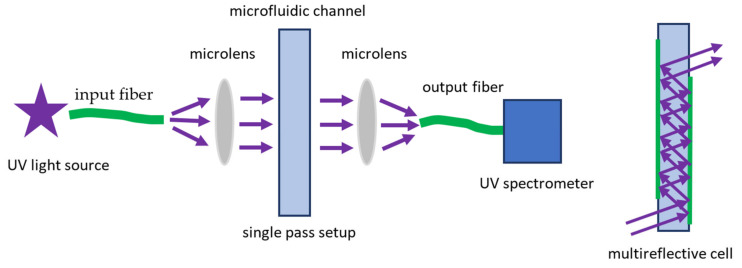
UV absorption detection in a microfluidic system (violet arrows—UV light rays).

It follows from the equation (1) that both relatively weak UV absorption of proteins and very short light pass lengths in a transverse detection scheme (Figure 5) limited by a width of a microfluidic channel contribute to the main disadvantage of UV absorption detection–low sensitivity. Different approaches are used to enhance sensitivity by increasing the optical pass length and minimization of optical losses: multireflection optical cells [181], optical fibers and bare photodetectors directly integrated in a microfluidic structure [182,183], microfibers with evanescent field absorption [184], multimode detection in a single device [185]. For bovine serum albumin (BSA), a limit of detection (LOD) as low as 1 ug mL^−1^ (15.2 nM) was achieved using an integrated detector [183] and even 10 fg mL^−1^ with evanescent field absorption [184].

Despite all methodological and technological improvements in microfluidic devices, UV absorption spectroscopy remains less sensitive and specific among other optical methods for protein detection due to its fundamental limitations. It is relatively simple, does not require expensive equipment, and could be a method of choice when high sensitivity and specificity are not necessary.

#### 3.3.2. MIR Spectroscopy

**Figure 6:**
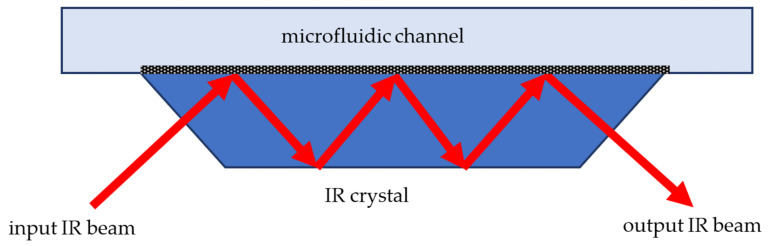
Measuring MIR absorption spectra using ATR setup (red arrows—IR light rays).

In the MIR region, the main challenge for spectroscopic detection in water solutions, including biological fluids, is strong water absorption [186]; therefore, a very thin layer of liquid in case of transmission IR spectroscopy (TM-FTIR) or, which is more preferable, an optical scheme based on attenuated total internal reflection (ATR-FTIR) should be used. The main element of an ATR accessory is an optically dense IR crystal with a high refractive index which is simultaneously transparent in a working spectral range. The IR crystal is installed into the wall of a microfluidic channel [187,188,189] or connected to the microfluidic chip in a separate flow-through cuvette [190], the surface of the crystal is in direct contact with a liquid analyte (Figure 6). An incident IR beam travels through the crystal and undergoes multiple total internal reflections, and an evanescent electromagnetic wave propagates in the liquid along the border. In regions of the spectrum where the liquid absorbs electromagnetic energy of the evanescent wave, the IR beam in the crustal is attenuated. The reflected beam is analysed with an FTIR spectrometer.

Various modifications of TM-FTIR, ATR-FTIR and other IR techniques including surface-enhanced infrared absorption spectroscopy (SEIRAS), infrared reflection absorption spectroscopy (IRRAS), vibrational circular dichroism (VCD), and microfluidic modulation spectroscopy (MMS) are extensively used in protein science. SEIRAS provides particularly high sensitivity due to the fact that vibrational absorption of molecules adsorbed on or present near nanostructured noble metal films may be enhanced by a factor of 10–1000 in magnitude [191]. SEIRAS is particularly useful for the investigation of membrane proteins [192]. The IR spectroscopy of proteins is an inherently label-free technology which gives it unique capabilities for structural analysis, investigation of molecular interactions and detection in a native conformational state. Some examples of IR spectroscopy applications in microfluidic devices for investigation of proteins are presented in the Table 4.

ATR FTIR technology is technically more complicated and expensive than UV absorption spectroscopy; it can only detect an optical signal from a very thin near surface layer of a medium, which makes it extremely sensitive to the adsorption of a protein layer on the surface of the ATR crystal [190]. Despite this obvious limitation, ATR FTIR is an indispensable approach for structural and conformational analysis of proteins or chemical imaging in microfluidic chips [187,188,190].

### 3.4. Protein Detection by Intrinsic Fluorescence 

Fluorescence spectroscopy is one of the crucial tools in biomedical research because of its much higher sensitivity and specificity in comparison with spectrophotometry. Fluorescence based optical techniques are widely used for protein detection and qualitative determination both in classical analytical instruments and microfluidic systems. In most cases fluorescence detection requires labelling when fluorophores are chemically attached to or incorporated into protein molecules. Fluorophores are specially selected molecules or molecular groups with a high quantum yield which produce strong and easily detectable fluorescence signals in the visible region, i.e., organic dyes (fluorescein, ethidium bromide, cyanine), fluorescent proteins (GFP, RFP, YFP), and quantum dots. Labelling provides excellent specificity due to the high affinity of a fluorophore to a specific protein, for example by using labelled antibodies [204].

From a practical point view, labelling is often an expensive and time-consuming process which should be avoided when possible. Moreover, labelling can change structural and functional properties of protein molecules. In this context, techniques based on intrinsic fluorescence are often preferable.

**Figure 7:**
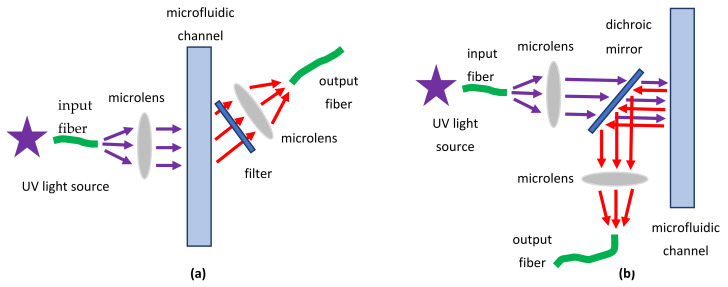
Protein fluorescence detection: orthogonal (**a**) and epifluorescence (**b**) setups (violet arrows—excitation optical radiation in the UV region, red arrows—fluorescence radiation in the visible or IR region).

In microfluidic systems with fluorescence detection, two types of optical setups are most widely used: orthogonal or angular detection, where the angle between excitation and fluorescence beams is 45 or 90 degrees, and epifluorescence detection, where both beams are parallel to each other (Figure 7). In the case of orthogonal detection, a narrow-band rejecting optical filter cutting excitation radiation is installed; in the epifluorescence setup, excitation and fluorescence radiation are separated by a dichroic mirror. The latter scheme is often used in fluorescence microscopy [205].

Excitation of intrinsic protein fluorescence requires a powerful monochromatic UV light source: usually a UV laser, e.g., the 4th harmonic of Nd:YAG laser emitting at 266 nm or deep UV LEDs are employed. UV lasers produce more intense and highly collimated beams but are bulky and expensive; deep UV LEDs provide much greater selection of working wavelengths; they are miniature and suitable for integration in microfluidic chips. Detection of the fluorescence signal can be realized at a single wavelength with a bandpass optical filter cutting excitation radiation and a highly sensitive photodetector, e.g., a photomultiplier tube (PMT) or avalanche photodiode (APD); spectrofluorimetric setups can be also used [205,206].

To increase the sensitivity and specificity of protein detection, instead of conventional spectrofluorimetry based on the measurement of the spectral intensity of a fluorescent signal, more advanced fluorescence techniques are often used in microfluidics: two-photon excited (TPE) fluorescence, fluorescence correlation spectroscopy, Förster resonance energy transfer (FRET), fluorescence lifetime detection, time-resolved fluorescence spectroscopy, microscale thermophoresis (MCT), nanoscale differential scanning fluorimetry (nanoDSF), etc.

Despite the long history of thermophoresis in various fields of science and technology, the microscale thermophoresis (MCT) method in its modern form appeared relatively recently, in 2010 [207,208]. NanoTemper Technologies put MCT on the market. Initially, the method was aimed to study the affinity of fluorescent-labelled macromolecules [209]. To analyse it, mixes of examined fluorescently-labeled molecules with various concentrations of non- fluorescent partner molecules are placed in capillaries. After registering the initial level of the fluorescence signal, an infrared laser (1475 nm) heats up the capillary area where the fluorescence signal change is detected. During heating, the temperature rises due to the “scattering” of the sample molecules from heated volume into neighbouring, colder areas. After the temperature rise, thermophoresis begins, and the sample reaches a stationary state within 25–30 s. After the infrared laser is switched off and heating stops, reverse diffusion is observed. Analysis of the obtained curves of fluorescence intensity allows for the calculation of the following parameters: the number of complexes, the rigidity of the structure and size of the macromolecule, as well as the tendency to aggregation [210,211]. Knowing the correlation between the number of complexes and the concentration of the ligand, it is possible to precisely calculate the dissociation constant Kd for the selected compound. Currently, MCT can be used to study affinity without using a fluorescent label but with intrinsic protein fluorescence due to the presence of phenylalanine, tyrosine, and tryptophan [212]. The MCT arrangement requires capillary analysis, which makes it easy to transfer this method to a microfluidic system. There is no data on the use of MCT (well as of NTA) to determine the affinity of macromolecules as a microfluidic chip. However, thermophoresis itself is already applied in microfluidic systems, for example, to separate the molecules [213].

The Nano differential scanning fluorimetry method is used to study the conformational stability of proteins during various manipulations. The principle is as follows: the protein in the solution is heated, and that leads to the unfolding of the protein [214]. During the heating process, intrinsic protein fluorescence is registered, mainly due to the presence of aromatic side chains of tyrosine and tryptophan. During the unfolding of the molecule, the surroundings of the fluorescent regions change as they interact with the solvent changing the fluorescence signal. Analysis of the change in fluorescence intensity under the influence of a temperature gradient makes it possible to calculate the so-called apparent melting temperature. Just like MCT, this method is available on the market. The sample is examined in capillaries, which makes it possible to implement it as a microfluidic chip. Moreover, the technical implementation of all the necessary conditions for the nanoDSF principle has been already used in a device for assessing the thermal stability of biomolecules based on a segmented flow microfluidic system [215].

Some examples of microfluidic devices for protein research with optical fluorescence detection are summarized in Table 5.

### 3.5. Refractometry in Microfluidics 

Refractometry methods of refractive index measurements are widely used in chemistry, biology, and medicine. Refractometry methods are very attractive for chemical and biochemical sensing due to the lack of need for labels. The main parameters, which can be obtained by these methods, are the presence of impurities in the solution and its concentration. Proteins were studied by refractometry methods for decades (see review [226]). Such research continues to this time [227,228,229,230,231,232,233,234,235].

The development of microfluidics has presented researchers with new problems. These are the miniaturization of refractometers to micrometer scale, the increase of their sensitivity due to refractive index change, and ensuring their compatibility with microfluidic devices. As a rule, these problems are solved on the basis of integrated optics. Let us now consider some refractometers for microfluidics.

Most of the refractometers for microfluidics are based on integrated optics devices with resonance properties: interferometers, ring and disk resonators, resonators on whispering gallery modes, etc. Spectral position of resonance of such devices depends on the refractive index of the environment. When the refractive index of the environment changes, the spectral shift of resonance takes place. The two most important parameters of the microresonator must be maximized to ensure the high sensitivity of the refractometer: the quality factor (Q-factor) of the resonance, which determines of resonance spectral width, and the slope between maximum and minimum transmission/reflection states [236]. 

Various microresonators with high Q-factors have been realized in recent years for refractive index measurements in aqueous environments [237,238,239]. Several resonant sensors for microfluidics have been demonstrated, based on microcavities coupled to integrated waveguides as the input and output ports. Silicon microdisks with moderate Q (of about 5000) have enabled the detection of fluid refractive index variations down to 10^–4^ for 10 femtolitres of surrounding fluid volume [240]. Polystyrene microrings (Q ≈ 20,000) have enabled the detection of glucose concentrations of 0.1% as well as the specific binding of biomolecules with low-mass coverage on their surfaces [241]. 

Many refractometers for microfluidics are based of Mach-Zehnder interferometers. They have ultrahigh sensitivity of 10^−4^–10^−8^ but require long interaction lengths—up to several centimeters [242]. The length can be increased considerably without the decrease of sensitivity using the liquid core waveguide in a Mach-Zehnder interferometer. As it was shown in [243] in this case, the sensitivity can reach 4 × 10^−6^ for the length of interferometer of 30 μm.

Fiber Bragg gratings are another kind of resonator structure with sensing capabilities: they rely on the spectral shift of the Bragg resonance when the refractive index of the surrounding analyte is changed [244,245]. The length of such a refractometer can be tens of micrometers and have a sensitivity of 4 × 10^–4^. 

A compact and integrated 50-μm long refractometer has been described, in which a microfluidic channel containing analyte forms the optical cavity of a Fabry-Perot interferometer [246]. For input and output of optical signals, optical fibers are used. At the butts of optical fibers, which are in the microfluidic channel, Bragg gratings are formed. They play the role of selective interferometer mirrors. The resonances of this interferometer are very narrow (less than 0.02 nm) and it enables the detection of refractive index variations down to 2 × 10^–3^.

Planar diffraction gratings are also used in refractometers for microfluidics, though they are non-resonant devices. Their advantage is in relaxed optical alignment in comparison with direct end-coupling into waveguides [247]. In [248], the polymer H-shaped microfluidic device with grating is described. In this device a reference fluid and analyte fluid could be passed over the grating under the same conditions and measured simultaneously. The sensitivity of microfluidic refractometers with gratings can reach to 4 × 10^–4^ [249].

Photonic crystals can be also used for refractive index measurements in microfluidic devices. As it was shown in [250], using computer simulation, a 1D photonic crystal with defects, consisting of four silicon plates and divided by air gaps, can be used for refractive index measurements of liquid analytes in near IR and THz spectral ranges simultaneously. The sensitivity of such photonic crystal in the near IR range is 275 nm/RIU (RIU: Refractive Index Unit) and in the THz range it is 424 GHz/RIU.

Some examples of refractometric microfluidic devices for detecting proteins, created over the past decade, are considered in Section 3.8.2.2 and Section 3.8.3.

### 3.6. Plasmon Resonance for Protein Detection

Today, the active development of biosensorics is observed due to the wide using of sensors for complex biochemical analyses both in the laboratory and at home. Using sensor technologies, it is possible to obtain qualitative and quantitative analysis in real time with minimal additional preparation of the analysed substance. Such an analysis, due to the high sensitivity, fast response, and low probability of biosensor error, in a short period of time can lead to obtaining preliminary useful information about the material under study.

Specific biochemical reactions are the basis for the formation of an analytical signal in the biosensor. For this, biochemical or biological components are used as receptors. Signals can be of thermal, electrical, or optical nature. It is the optical biosensors that allow the detection of a very small amount of a substance and are easily adaptable to the analysis of various biological and chemical objects that have received the greatest recognition in recent years [251]. One of the main parameters of biosensors is their sensitivity. It can be increased by creating innovative materials and ensuring effective communication between sensor components.

To date, the appearance of a significant number of publications on the detection method based on plasmon resonance is of great interest to scientists interested in such sensors [252]. The basic principle of operation of such biosensors is plasmons—a special type of charge density waves arising from the interaction of photons with elementary excitations of the medium and propagating along the metal/dielectric or metal/vacuum interface. Plasmons can be surface or localized. The latter are manifested in the case of interaction of light with metal nanostructures, the size of which is less than the wavelength of the incident electromagnetic radiation. The waves arising during the interaction are strongly localized at the interface between the media, which leads to their high sensitivity to any changes in the boundary conditions. The possibility of using this property for detecting especially low concentrations of the investigated substances was the main reason for the widespread use of SPR sensors [253].

#### 3.6.1. Sensors Based on SPR

Most SPR sensors for detecting proteins use a metal-dielectric interface as the simplest scheme for exciting surface plasmons. Using Maxwell’s equations, it is possible to show the existence of only one propagating mode of the electromagnetic field with specified boundary conditions. This mode is the surface plasmon. One of the peculiarities of metals is the negative value of the real part of their complex permittivity in the UV, visible and part of the IR spectral range. The complex part of the dielectric constant can have relatively small values in the previously considered range for metals such as gold and silver. This indicates a weak absorption in metals, which led to the widespread use of gold and, more rarely, silver films in SPR-based biosensors. The high frequency of gold usage is associated with its high stability in saline buffer solutions. At the same time, despite the better optical properties of silver in comparison with gold, it rapidly degrades when exposed to external conditions. These negative effects can be eliminated by coating the silver with a dielectric layer or using a two-layer gold/silver structure.

**Figure 8:**
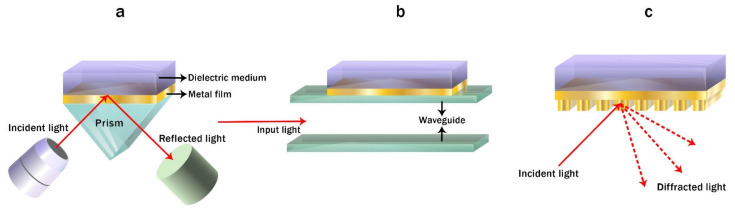
The excitation of surface plasmon resonance by different light coupling methods for SPR biosensing, including (**a**) by Kretschmann configuration, (**b**) by the optical wave-guide coupling, and (**c**) by grating coupling.

The surface plasmon is a transverse wave; therefore, its electric field vector is perpendicular to the metal/dielectric interface and to the direction of its propagation. Generally, in SPR sensors, a surface plasmon-polariton can be excited by a light wave. However, the SPR propagation vector is much larger than the wave number of a light wave in a dielectric medium; therefore, surface plasmons cannot be excited by direct illumination. To excite a surface plasmon, it is necessary to match the projection of the wave vector of the incident radiation parallel to the interface and the wave vector of the surface plasmon. This can be achieved by amplifying the incident light in several different approaches including prism, waveguide and grating coupling (Figure 8).

In many SPR sensors, surface plasmons are excited using the Kretschman prism scheme [254,255], which has become widespread due to its simple implementation and the possibility of using methods with various types of signal modulation. The basis of this method is the transmission of exciting radiation through a prism with a high refractive index; when reflected from the base of the prism, an evanescent wave is generated. Excitation of the plasmon occurs due to the penetration of this wave into a thin metal film deposited on the base of the prism (Figure 8a). Another way to efficiently excite a surface plasmon is to use a waveguide with a thin metal film on its lateral surface (Figure 8b) [256]. The light passing through the waveguide reaches the region with the metal film and the evanescent wave will excite the surface plasmon at the outer boundary of the metal layer. For the third method, a scheme of radiation propagation from a dielectric medium onto a metal grating is used [257,258,259]. The diffracted radiation can excite a surface plasmon if the projection of its wave vector, parallel to the surface of the grating, is equal to the wave vector of the surface plasmon (Figure 8c).

#### 3.6.2. Sensors Based on LSPR

In recent decades, the tremendous interest of scientists in nanotechnology has led to excellent results in the development of new methods for the creating and assembly of nanomaterials. The obtained results have been widely applied to various fields of science and industry, including the protein sensor direction of research [260]. In particular, special attention has been focused on metallic nanomaterials due to their unique optical properties in the field of plasmonics. Plasmonic nanoparticles obtained from noble metals possess high stability, high surface energy, excellent biocompatibility, and could lead to strong signal amplification, which has made them ideal candidates for use in the new generation of biosensors [252,261,262].

Localized surface plasmon resonance is a type of SPR arising from collective or non-propagating oscillations of conduction electrons when the electromagnetic field is limited by metallic nanostructures. In this case, strong quenching of light will lead to both partial absorption of incident photons and also their scattering in different directions, which can lead to the accumulation of polarization charges on the surface of metal nanostructures. When the absorption spectrum is recorded, a strong peak will be observed due to the excitation of the LSPR plasmon nanostructure by the incident light. The height of the LSPR peak and the corresponding wavelength will be sensitive to morphology, size, material, and distance between adjacent nanostructures, as well as to the environment around the plasmonic nanostructure, which can be used as a sensitive medium [263,264]. The study of the dependence of the absorption and scattering efficiency on the parameters of three gold nanostructures - nanospheres, silica nanoshells and nanorods, calculated by the discrete dipole approximation and using the Mie theory, showed the effect of the nanoparticle size on the resonance wavelength, the ratio of scattering to absorption, and the extinction cross section [264,265]. For example, the best photoabsorption is observed in nanorods with a high aspect ratio and a small radius, while nanorods with a high aspect ratio and a large radius exhibit the highest scattering contrast and can be used for imaging. Also, changing the chemical and physical properties of plasmonic structures can help adapt and tune the LSPR properties of the plasmonic nanostructure from ultraviolet (UV), visible (VIS) to near infrared (NIR) regions in the optical spectrum [266]. Depending on the field of application of the sensors, various metal nanostructures can be used in them, such as nanospheres, nanorods, nanoshells, nanowires, nanoprisms, etc. 

#### 3.6.3. Microfluidics-Based Plasmonic Sensors

Significant innovations in the development of inexpensive methods of nanoproduction on large areas and the development of nanolithography technologies have made it possible with a high degree of controllability to fabricate various plasmonic metal nanostructures integrated into microfluidic systems [267]. Considerable progress has been made in the development of unique and at the same time convenient, automated, and portable microfluidic plasmonic biosensors that meet the special requirements of clinical practice and use at the point of care. However, the development of micro/nano-optics continues and new advances in plasmon resonance methods and optical components are gradually emerging.

LSPR-based sensors have become possible using not only colloidal particles, but also microchip-based substrates that are miniature with high sensitivity and reproducibility, and can be integrated with other sensitive components. Plasmonic sensors use both solutions of colloidal nanoparticles and their arrays. One of the main advantages of using an array of nanoparticles in detectors based on plasmon resonance is the absence of the phenomenon of clusters and agglomeration of nanoparticles. This allows for better reproducibility of results, and the nanostructured array can be easily integrated with microfluidic technology for multiplex and ultrasensitive analysis. Examples are microfluidic biosensors with gold nanorods. As an alternative highly sensitive metal structure, subwave pattern nanoholes can be used. The presence of one nanohole in optically thick metal films can be accompanied by an increase in the localized electric field near the edge of the hole, but the transition to periodically located holes will be accompanied by the appearance of surface plasmon polaritons. The existence of LSPR without the presence of SPP can be ensured by passing to a non-periodic array of nanoholes. The absence of bulky circuits using prisms for the presence of SPR made it possible to easily switch to microfluidic plasmonic biosensors based on template nanoholes or cavities with the possibility of high-throughput multiplex analysis with high sensitivity.

In addition to SPR/LSPR probing methods, novel approaches to plasmon biosensing have emerged. Thus, the transmission configuration method (T-LSPR) is based on the ability of the LSPR to generate a non-propagating plasmon mode when using a thin metal film on the disconnected template [268]. The T-LSPR sensor device consists of a sensor chip functionalized with an aptamer based on DNA specific to the antibiotic tobramycin on gold nanoislands (NI), deposited on a glass slide and coated with fluorine doped tin oxide (FTO). Such a T-LSPR sensor can detect in real time tobramycin in a buffer with a concentration of up to 0.5 μM [269]. It is also possible to create special microfluidic systems with SPR sensing capabilities aimed at detecting well-defined biomarkers. Another optical technique based on spatial control of local differences in incident light reflectance caused by interaction with the analyte through prism coupling is SPR Imaging, first introduced by Benno Rothenhuisler and Wolfgang Knoll in 1988 [270]. At the beginning of the development of this method, multi-analysis was realized by estimating a two-dimensional picture of the intensity of reflected radiation, however, through serious improvement of the method, data on angle, wavelength, phase, and polarization, obtained with SPRi sensors based on incident light, began to be used for analysis. The use of the SPRi tandem and microfluidic system offers real-time detection capability with subnanomolar sensitivity, and the immunoreaction can be detected and quantified in about a few minutes [271].

#### 3.6.4. Protein Detection by Surface-Enhanced Raman Spectroscopy 

Surface-enhanced Raman scattering is based on the capability of metallic nanostructures (nanoparticles) to concentrate electromagnetic waves by exciting surface plasmons and amplifying otherwise weak Raman scattering signals. It provides a strong mean to overcome the low efficiency of the ordinary Raman spectroscopy compared with fluorescence and FTIR absorption spectroscopy. SERS enables detection of very small amounts of analytes, even single molecules [272]. In terms of protein detection, this technique not only makes the testing of native biological samples for specific biomarkers of various deceases on a single molecular level p(liquid biopsy) possible, but offers valuable information about molecular vibrational and rotational transitions associated with structural properties and conformational changes of proteins in complex mixtures [273,274]. It improves detection speed and requires extremely low sample volumes. Both label and label-free methods are employed for protein detection; the label-free approach gives multiple advantages already discussed in the beginning of this chapter but could be applied to only a limited number of protein molecules containing chromophores. Novel highly efficient SERS substrates are developed to enhance intrinsic Raman signals and create new techniques for label-free detection [275]. In the recent decade, label-free SERS have been extensively used in biological and biomedical applications such as drug monitoring, early cancer diagnosis, pathogen identification or analysis of cellular mechanisms [276]. 

Further enhancement of Raman signals can be achieved via chemical functionalization of SERS substrates [277,278], i.e., with alkane thiol-based molecules [279], ε-caprolactone [280] or antibodies [281,282,283]. This strategy allows for the achieving of increased sensitivity and improved specificity to SERS-based protein sensing devices. Some examples of label-free SERS protein detection in microfluidic platforms, including biosensors with chemically functionalized SERS substrates [281,282,283], are presented in Table 6.

SERS and other plasmonic-based optical techniques are among the most sensitive, often detecting single molecules of protein biomarkers. Moreover, it is suitable even for in vivo diagnostics. The major drawback of this method is that it requires laser excitation, specially prepared substrates, and expensive Raman spectrometers, but recent developments of this technology make it more affordable for implementation in real clinical practice. 

### 3.7. Application of Diffusometric Methods for Protein Characterization

#### 3.7.1. Dynamic Light Scattering 

In the second half of the 20th century, the dynamic light scattering (DLS) or photon correlation spectroscopy (PCS) method became widespread as a way to analyse the diffusion of macromolecules in solutions [296,297]. The DLS allows for the determination of the diffusion coefficient of colloid particles in a solution by analysing the correlation function of fluctuations in the scattered light intensity [298]. The size of a particle is calculated with a diffusion coefficient, and it allows the detection of particles ranging from 1 to 1000 nm in the protein concentration range from 0.1 mg/mL. Multi-angle detection makes it possible to determine not only the size of the particles, but also their form factor, and, with a certain extent of accuracy, of their concentration in the solution. Nowadays there are many commercially available systems for various purposes; the most famous is the Zetasizer (Malvern Panalytical, Malvern, Worcestershire, UK) series. Along with DLS, the device implements the possibility to measure the zeta potential of particles in solution. It is an important characteristic of macromolecules that determines the stability of the colloidal system. This method is quite simple to execute and can be implemented in a microfluidic system for detecting particle size [299,300]. Described microfluidic systems with built-in DLS includes in its design a channel that provides the microflow of the test sample, fiber-optic light guides which illuminate the sample and record the signals, and a system mixing the sample immediately before measuring DLS. It was shown that such systems register particles from 10 to 800 nm in organic and aqueous solvents, the particle concentration in the test sample is 0.1 mg/pL [300]. We can use fiber-optic light guides to implement a multi-angle DLS registration scheme to measure the qualitative parameters of the distribution intensity and to assess the volume ratio of particles in the colloid. 

#### 3.7.2. Nanoparticle Tracking Analysis 

Nanoparticle tracking analysis (NTA) as well as the DLS is a diffusometric method for colloidal system research. It can detect particles from 30 to 1000 nm and determines the particle concentration in the solution in the concentration range from 10^7^ to 10^9^ particles/mL depending on a sample type [301]. NTA combines laser light scattering microscopy and a camera that registers movement of diffraction spots caused by light scattering on particles in the examined field. The volume of the examined field for NanoSigth LM10 (Malvern Panalyticall, Malvern, Worcestershire, UK) is approximately 100 × 80 × 10 μm [302]. Analyzing the registration, the following parameters are calculated: the speed of geometric centers of the diffraction spots and the diffusion coefficient of individual particles of the colloid. The collected data makes it possible to characterize the size distribution of colloid particles. At the same time, it is possible to determine both the total concentration of the particles and the concentration of particles in the selected size range. The disadvantages of the method include a relatively narrow dynamic range and limited sampling (of the particles in the examined field). Currently, there are no examples of NTA implementation in microfluidic systems. We tend to think that the basic NTA arrangement—a flow camera, an illumination source and an optical system, an optical window, and an external registration camera—can be implemented as a microfluidic system. Collected data may be difficult to process, but there is software for live particle tracking called PyNTA: Python Nanoparticle Tracking Analysis v. 3.2 (open-source software https://pynta-python-nanoparticle-tracking-analysis.readthedocs.io/, accessed on 2 December 2021) [303].

### 3.8. Optic Components in Optofluidics for Protein Detection 

Optofluidics combines the advantages of optics and microfluidics; the integration of optical elements into a microfluidic chip allows one to implement optical methods for detecting, manipulating (trapping, sorting and aggregation), and analyzing biomolecules. The advantages of the approach include the following: (i) miniaturization (minimization of bulk optics outside the chip); (ii) the ability to receive a signal from small volumes of analyte, and as a result, a decrease in detection thresholds; (iii) increasing the sensitivity; and (iv) reducing or eliminating alignment problems with optical components. However, this increases the complexity of manufacturing, which can also affect cost and reproducibility, and it may be necessary to use combinations of technologies. Excellent reviews on optofluidics in general (for cytometry, cell biology, protein and nucleic acid detection, and chemical analysis applications) are given in [133,304]. Here we focus on optical detection of proteins using optofluidic chips with integrated optical elements. In some cases, examples of devices tested with organic dye solutions, traditional biomarkers for proteins, are given.

**Figure 9:**
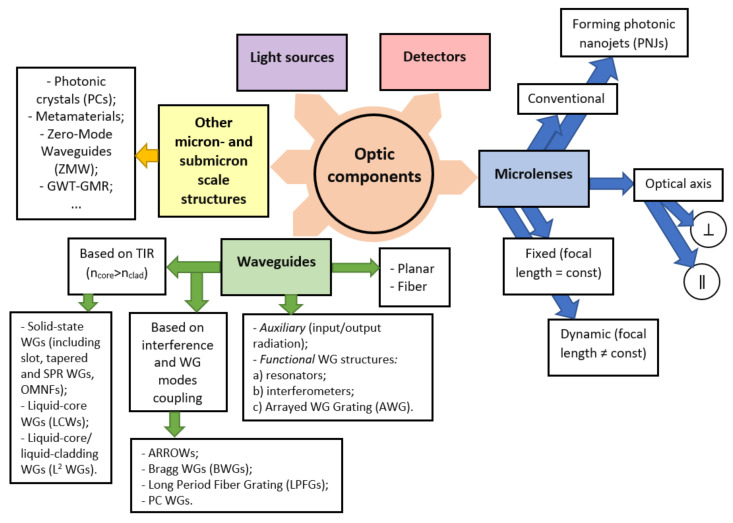
Optic components in microfluidics. ‖—parallel, ⊥—perpendicular.

In general, the component base of optofluidics can be represented as shown in Figure 9. Optoelectronic devices such as light sources [305] and detectors can be integrated into a chip [306]. In addition, the concept when the analyte is an active radiation source is implemented in optofluidic lasers. For example, in [307] the channel with the analyte and the gain medium (Rhodamine B solution) surrounded by reflecting surfaces (Fabry-Perot resonator) is described. Based on a modification of the turbidimetric inhibition immunoassay (TIIA) method, the concentration of the target IgG is determined by the intensity of laser emission. This approach allows for the increasing of the detection sensitivity and dynamic range.

**Figure 10:**
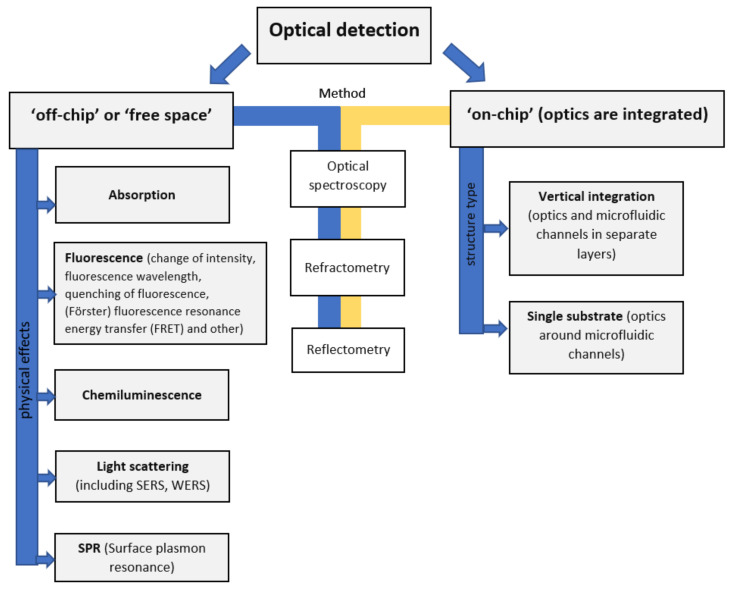
Optical detection in microfluidics.

However, it is more common in optofluidics to integrate optical elements to control the propagation of radiation through a section with an analyte: focusing and collimating light (microlenses), redirection (micromirrors) and localization of radiation in or near a cavity with an analyte (waveguides, micromirrors) for more effective interaction of optical radiation and analyte, for miniature implementation of the sensor optical scheme (for example, for refractometry). In addition, fluid flows can have different refractive index profiles and act as lenses [304]. Depending on the physical effect or the detection method (Figure 10), chip architecture is implemented. It should be noted that the classification of methods and components (Figure 9 and Figure 10) is given in general terms, since often different methods can be mixed, or components used in different aspects are used. We discuss the various components in more detail below. Table 7 presents the main examples of the implementation of optofluidic devices considered in this section.

#### 3.8.1. Microlenses

Microlenses as elements integrated into the chip perform the same functions as external bulk optical elements: focusing or collimating light flux. However, integration into a chip makes it possible to fabricate them in a miniature fashion, and they are more resistant to external influences, to limit the radiation collection area and, as a result, to increase the detection sensitivity. The specificity of microfluidics is that the fluid flow in the channel can have a gradient of the refractive index n in both the transverse and longitudinal directions, which affects the propagating radiation [304]. Therefore, liquid flows can form microlenses or, on the contrary, scattering media, which can be both a disadvantage (additional radiation losses) and an advantage (application for detection, reconfiguration of the optical path, etc.).

Microlenses can be formed by solid media [308,331], soft deformable surfaces [332], and liquid-liquid interface [333]. Changes in the parameters of such microlenses can be carried out by external influence (pressure, heating, electric or magnetic field) or by liquid flows inside the chip. Accordingly, the focal length can be constant (fixed lens) or variable (dynamic lens). The microlens optical axis either lies parallel in the plane of the chip (II) or perpendicular to it (Ʇ). It is also possible to subdivide microlenses into additionally assembled and self-aligned in terms of their positioning on the chip during the manufacturing process [334]. Additionally, assembled lenses require alignment, as self-aligned lenses are immediately integrated into the chip at a fixed location and can be performed in a single technique with the chip [335]. Lenses, in the formation or control of the parameters of which the liquids in the chip are involved, are called optofluidic microlenses. According to the operation principle, liquid microlenses in a chip can be divided into the interfacial deformation lens, refractive index modulation (gradient index) lens, the liquid-crystal based lens and the diffractive lens (for example, the Fresnel zone plate—periodic microstructure, filled with liquid) [332]. Air-gap walls inside the chip can be used as mirrors [308] if the condition of total internal reflection is satisfied. Control of such mirrors is possible if the air gap/channel is filled with liquid, since by changing the ratio of refractive indices at the channel/substrate interface, cancellation of total internal reflection for a fixed angle of incident radiation is achieved [335].

Let us give some examples of the use of microlenses for detecting proteins. In [331], 2D planar lenses (fixed, II axis) were manufactured using CO_2_ laser ablation in PMMA substrate. Lenses focus the exciting radiation into a microfluidic channel, and fluorescence is collected by an orthogonally positioned photodetector. The presented platform allows the determination of cardiac biomarker Troponin I by applying standard fluoroimmunoassay technology with a limit of detection (LOD) of 0.08 ng/ml. In [308], 2D planar self-aligned lenses (fixed, II axis) are used to collimate radiation through six microfluidic channels for absorption measurements. Micro mirrors made of air gaps keep scattered radiation in the measuring area and prevent cross talking. A feature of the chip is the configuration with parallel (“multiple path”) absorption measurements with different optical lengths for one analyte (the multiple path photonic lab on a chip (MPHIL)), which makes it possible to simplify and accelerate measurements and work in the absorbance vs. concentration linear range. The device has been successfully tested with five different proteins: lysozyme (HEWL), glucose isomerase (D-xylose-ketol-isomerase (GI)), *Aspergillus* sp. lipase L (BLL), *B. cereus* formamidase (FASE) and dihydropyrimidinase from S. *meliloti* CECT41 (DHP). 

The previously described variants of microlenses can be classified as conventional lenses for which the focusing area (optical resolution) is limited by aberrations [336] and the diffraction limit. An alternative is the approach using mesoscale dielectric particles of various shapes (spheroids, cuboids, cones, pyramids, trapezoids, hemispheric shells, and others) [337]. Such objects form photonic nanojets (PNjs), i.e., areas of strong local concentration of optical radiation near the shadow-side surface of a particle with a localization size of 1/3–1/4 radiation wavelength (in the cross section), which is less than the diffraction limit of a conventional lens. PNJs are applied for the enhancement of Raman signals [338], for imaging of nano-features and adenoviruses, for resolving the structures of subcellular organelles [133], for nanoparticle trapping [339], and for nanopatterning [340]. In [309], dielectric microspheres in a microwell array template for immunodetection of biomolecules immobilized on Au NPs in buffer are used. An increase in the fluorescence signal for the detection of biotin and mouse IgG by a factor ∼40 relative to the signal without microspheres is shown. In [310], using a monolayer of latex microspheres with 2 μm diameter the cellular protein Annexin A5b labeled with the Cyanine 5 (Cy5) fluorescent dye was detected by fluorescence correlation spectroscopy (FCS). It is reported that the method allows one to determine the concentrations of substances in the picomolar range, and to assess molecular parameters (photophysical blinking, diffusion coefficient, relative hydrodynamic radius) without expensive optics.

The use of PNjs is a good solution for signal amplification in fluorescence detection methods. However, this approach is characterized by the same drawbacks as for fluorescence detection methods in general: the need for target labeling and the presence of voluminous external equipment (spectrometers, etc.).

#### 3.8.2. Waveguides

**Figure 11:**
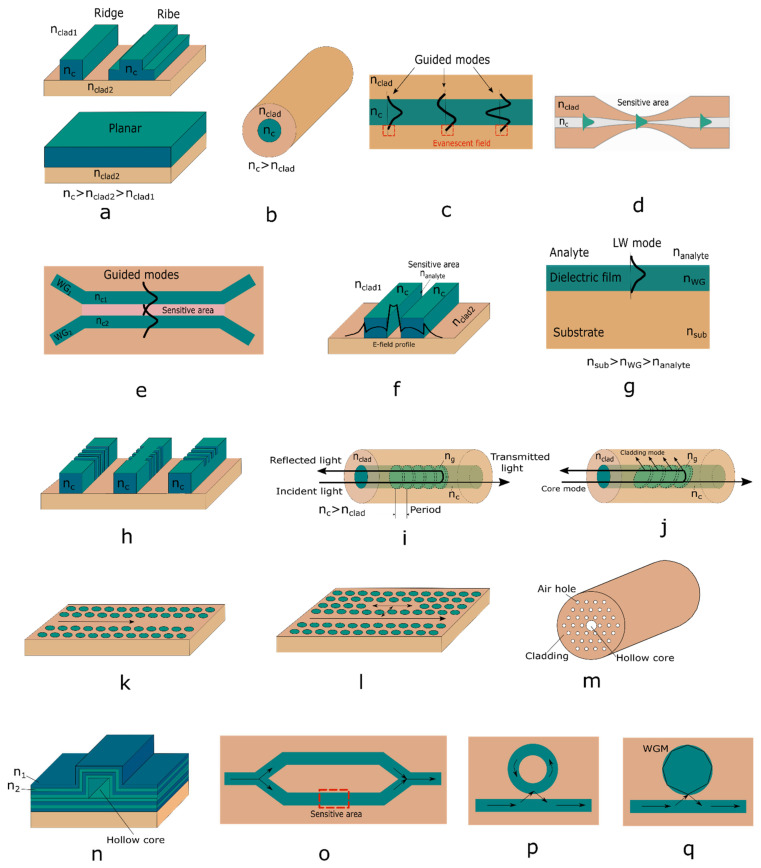
Optical WGs and WG structures in microfluidics. TIR-based WGs: (**a**) rectangular WGs (planar, ridge, rib); (**b**) fiber; (**c**) guided WMs in planar WG; (**d**) tapered fibers; (**e**) coupled waveguides (CWGs); (**f**) slot WGs; (**g**) leaky WGs (LWs). RI-modulated WGs: (**h**) Bragg grating WGs; (**i**) FBG; (**j**) TFBG; (**k**) PC-based WG (PC WG); (**m**) h-PCFs; (**n**) ARROWs. Resonance WG structures: (**l**) PC cavities with coupled PC WG; (**o**) waveguide-based Mach-Zehnder interferometer (MZI); (**p**) microring resonator; (**q**) microdisk resonator with whispering gallery modes (WGM)).

Optical waveguides (WGs) are a well-known technology for directional transmission of optical radiation through small objects (from hundreds of microns to tens of nanometers). Therefore, they are suited to integrate with microfluidics. The use of optical fibers (circular cross-section WGs, Figure 11b) inside the microfluidic chip limits its miniaturization (for example, due to the minimum bending radius of conventional fibers), therefore, rectangular WGs (planar, ridge, rib et al, Figure 11a) are more often integrated into the chip. 

The most common use of optical fibers is the delivery [341] and collection of radiation in the immediate vicinity of the microfluidic channel with the analyte, for example, for recording absorption and luminescence spectra with external equipment [308,342]. In refractometric techniques, the sensitive area is often made using rectangular WGs or WG structures. A detailed overview of the different physical methods and WG structures for biosensors is given in [147]. Since various types of WGs can be used in almost any method of optical detection, in this section we present a classification by WG types and give examples for protein detection. The main types of discussed WGs are schematically shown in Figure 11.

##### 3.8.2.1. TIR-Based WGs

One of the ideas for applying integrated WGs is to increase the interaction of radiation with a liquid while maintaining the radiation directivity, since due to optical confinement, light propagates along the channel with the liquid, while in the free-space configuration, the volume of interaction is limited by the cross section of the channel and the beam light (for example, in methods based on measuring the absorption and luminescence of the analyte). This is especially important for small volumes of liquid when the background luminescence and other optical noise are comparable to the signal levels from the analyte. Optical confinement (waveguide effect) can be realized in structures with effective multiple reflection in a certain volume, which leads to the formation of a stable electromagnetic field distribution called the waveguide mode (WM). This is achieved by specific profiles of the refractive index in the structure cross section [343].

A common approach to creating optical confinement in a WG is the total internal reflection (TIR) effect, which occurs at the interface between the core (c) and cladding (clad) under the condition n_c_ > n_clad_. In this case, the field of the WM is localized in the core, but also exists in the cladding in the form of an evanescent wave (Figure 11c) which allows interaction with the external influence while maintaining localization in the core, i.e., the ability to be directed (guided WMs). WMs that do not meet the TIR condition are called leaky WMs and have large losses, because they are scattered and absorbed in the cladding (external environment). Therefore, they are not used for directional transmission of radiation, but have found application in sensors.

Typically, WGs consist of a solid cladding and core or only a solid core (solid-state WGs). In the second case, the environment plays the role of a shell. Both the cladding and the core can be structured to form different types of modes. The influence of the analyte (by changing the optical properties near the core/cladding interface) occurs through the fields of guided WMs in the cladding. Tapered fibers [344,345] (Figure 11d) and planar WGs [346] are common examples of this approach for fluorescence detection, absorbance detection and refractive index (RI) detection [342]. In a microfluidic chip, an optical WG can have a liquid core (channel with an analyte in a solid substrate—Liquid-core WGs (LCWs)) or even be fully realized in a liquid medium (Liquid-core/liquid-cladding WGs (L^2^ WGs)) [133]. When interacting through the cladding, only the near-surface layer can be measured, since the penetration depth of the evanescent field into the cladding is several hundred nm. If the analyte is the WG core, then volumetric sensitivity is achieved.

Coupled waveguides (CWGs) are WGs located close enough to each other so that electromagnetic coupling is realized between them due to the overlapping of the evanescent wave fields (Figure 11e). CWGs are applying as a sensitive element because the coupling depends on the optical parameters of the medium between the WGs. Electromagnetic coupling is also used to exchange radiation with other structures in the chip.

In [313], a fiber light-coupled optofluidic waveguide (FLOW) immunosensor for the detection of tumor marker p53 protein is presented. The liquid-core capillary is connected to the fiber, and a dumbbell-shaped microstructure (tapered sensor) is formed in the narrowest part of which detection is carried out. In the sensitive region through the capillary wall (2 µm) and the fiber (diameter 5.6 µm), different modes propagate which interfere with each other. An RI change in the presence of p53 near the capillary wall introduces a phase shift, which leads to a shift in the fiber transmission spectrum. This configuration allows for the applying of optical fiber both as a sensing element and as a radiation carrier connecting with external equipment. The sensor showed high sensitivity, linearity, and specificity.

Slot WGs can be considered as a variant of enhancing the interaction through the field outside the core. Slot WGs consist of two solid-state strip WGs with a submicron (hundreds of nm) gap (Figure 11f). Due to the penetration of an evanescent wave outside the core during TIR, both WGs form a mode with a field concentration in the gap. Structuring (the creation of periodic and aperiodic structures) slot WGs allows one to enhance interaction or to expand the detection methods. In [314] the detection of hemoglobin, globulin and BSA protein in aqueous analyte by slot WG with grating with sensitivity of 600 nm/RIU and Q factor of 9650 (refractometry) is described. Slot WGs are also used as nanophotonic traps for nanoparticles (NPs) and biomolecules [347].

Combinations of different physical effects can increase sensitivity. It is typical to use plasmon resonance in microfluidics, in particular, plasmon WGs, both separately and in combination with other approaches. The SPR concept is discussed in more detail in the Section 3.7. In [315], the increase in interaction efficiency is achieved due to the greater fraction of evanescent fields of the WG and the local surface plasmon resonance (LSPR). The authors investigated optical micro/nanofibers (OMNFs) and optimized the diameter of these fibers for biosensing. OMNFs are optical fibers with a decrease in thickness down to subwavelength values (for the current wavelengths of transmitted radiation), while the evanescent field outside the core increases [348]. When NPs are deposited on OMNFs, the evanescent wave excites the collective oscillation of the conductive electrons in NPs, i.e., LSPR. Changing the medium RI around OMNFs leads to a modification of the transmission (absorption) spectra. In [315] the high sensitivity of such sensors for streptavidin with a LOD of 1 pg/mL is shown. [349] presents an overview of the applying of nanofibers in miniaturized analytical systems for the diagnosis of cancer, including cancer marker proteins detection.

Leaky waveguides (LWs) with leaky WMs are used in biosensing as a WG layer on a substrate (sub) with n_analyte_ < n_WG_ < n_sub_ (Figure 11g). The following types of LWs can be distinguished by the profile n in the cross section: symmetric and asymmetrical LWs [350]. For the asymmetrical LWs, light is confined in the dielectric film (WG) by TIR at one interface and partial reflection at the other interface. By the composition of the layers: antiresonant reflecting optical waveguides (ARROWs; several dielectric layers on the substrate), metal-clad leaky waveguides (MCLWs; metal layer between the substrate and WG), leaky lossy waveguides (LLWs; WG layer with large losses on the substrate), leaky waveguide gratings (LWGs; WG grating on the substrate) and diffraction-based LW (substrate and dielectric layer with (n_WG_-n_analyte_) ≤ (0.005)). In all cases, the WG layer has n_WG_ < n_sub_. Symmetric ARROWs (also called hollow core WG or liquid core WGs) are discussed in Section 3.8.2.3 because they do not use TIR. The input and output of radiation is usually performed through a prism with an RI matching oil to provide the required TIR angles at the liquid/WG interface. The reflected signal in the form of an angular intensity distribution has a resonance dip. Full width at half maximum (FWHM) of the resonance for LWs is significantly better than for SPR, therefore small shifts of the resonance can be identified. The metal film in MCLWs as a reflector can separate the WG from the substrate to increase the interaction path on the one hand and to increase the resonance dip due to losses on the other [351]. LW with a dielectric coating in the form of a porous film that traps biomolecules can increase the sensitivity to refractive index changes by 8–10 times [350]. In [316], diffraction-based LWs with chitosan waveguides are studied. The sensor design is very simple, a few microns thick hydrogel film on a glass substrate. The LOD of 1.9 × 10^−6^ ± 1.3 × 10^−6^ RIU and RI sensitivity of 125.5 ± 3.8 deg/RIU on the eight devices are reported. The authors also demonstrated that diffraction-based chitosan LWs can be used for monitoring analyte binding in the presence of 750 μM BSA.

##### 3.8.2.2. RI-Modulated WGs and Resonance WG Structures

Structuring (periodic modulation of n) WG parts makes it possible to increase the effect of analyte variation on the optical signal due to the coupling of different mode types, which affects the spectral characteristics of such structures (shifts of resonance bands). In fact, structured WGs are a particular case of photonic crystals (PCs) with waveguide properties. The waveguide effect can be realized regardless of the PC, for example, by TIR, or due to the properties of the PC (high reflectance in a certain spectral range).

PC-based WGs (PC WGs) are available in planar (Figure 11h,k) and fiber geometry (Figure 11i,m). In a fiber, periodic modulation n is possible in the longitudinal direction (along the core, for example, fiber Bragg gratings (Figure 11i) as a special case of 1D PC), as well as in the transverse direction (in the cladding)—photonic crystal fibers (PCFs), while the core can be solid and hollow (h-PCFs, Figure 11m). In the latter case, the fiber can act as a microcapillary. The waveguide properties of PC WG are due to “band gap” (maximum reflectivity) in the transmission spectrum of such structures. If the periodicity is violated (the appearance of a defect—a change in the geometry, dimensions, or in of one of the layer/holes), an additional spectral band appears in the photonic band gap.

In fiber Bragg gratings (FBGs) with a modulation period <1 μm, core modes interact with each other, forming a backward reflected signal. Usually, gratings are written in the core of the clad fiber. Therefore, this grating interacts with the environment only through thermal and mechanical fields. For RI measurements, part of the cladding is removed (e.g., by chemical etching) or special refractive index profiles (e.g., tilted FBGs-TFBGs [352]) are used. TFBGs allow the formation of cladding modes that interact through an evanescent field with the external medium (Figure 11j). The TFBGs transmission spectrum contains many resonance dips. In the planar version, the cladding is usually missing. Combinations of different methods are also used to increase sensitivity. Arrays of FBGs can be used for multiparameter sensing. In [317], pH, temperature, humidity, gas concentration, light intensity and BSA protein concentration with a sensitivity of 5 pm/μg/ml were measured using nanomaterial--coated multiplexed FBG sensors. In [318] TFBGs with nanometric silver coating for urinary protein detection was demonstrated. Proposed sensor construction allows the combination of «cut-off» (cladding modes near the cut-off) and plasmonic resonance methods. Differential amplitude measurement between both of resonances increases sensitivity and minimizes the effect of ambient temperature. An LOD of 1.5 × 10^−3^ mg/mL and sensitivity of 5.5 dB/(mg/mL) was achieved.

Long period fiber gratings (LPFG) with a modulation period of tens to hundreds of micrometers are easier to manufacture, and mode coupling in the cladding and core allows for increased interaction with the external environment. The resonant properties are displayed as a dip in the WG transmission spectrum. In [319], LPFGs in double cladding fiber with W-type RI profile were written and coated with nanometric layer of graphene oxide. The structures support the mode transition phenomenon - the interaction of cladding modes (outer cladding with a large n) and core modes. A LOD of 0.15 ng/mL and wide working range of 1 ng/mL–100 μg/mL was obtained.

In two-dimensional PC, waveguides (Figure 11k) and microcavities (Figure 11l) can be formed, for example, as with PC cavities, which are used for detecting proteins [353]. In [320], microcavities with coupled waveguides are formed in a 2D silicon-based PC. Three protein markers (Table 7) in plasma from pancreatic cancer patients were detected applying microcavities of two types (without and with nanoholes). It showed a 50 times increase in sensitivity compared to ELISA.

The improvement of optofluidics based on WGs with an increase in sensitivity and detection thresholds are resonant structures such as microresonators (microring (Figure 11p), microdisk (Figure 11q), microsphere and microtoroid resonators forming a whispering gallery mode (WGM)) and interferometers (Fabry-Perot (FPI), Michelson (MI), Mach-Zehnder (MZI, (Figure 11o)), Youhg’s (YI)) [133], in which, with comparable dimensions, a multiple pass of radiation, characterized by a quality factor Q, is realized. In fact, this is a way to increase the “path” of interaction while maintaining the device miniaturization. The development of high-Q devices is a challenge for researchers. An example of a disk resonator in which whispering gallery modes (WGM) are excited through a WG coupling (a WG with a small gap from the disk) is given in [354]. The structure is tested with Cargille fluids (series AA) for n range from 1.296 to 1.363 at 1550 nm. The sensitivity of 40 nm/RIU and the quality factor of 2 × 10^5^ is shown, which are typical values for this kind of device, and can be used for optofluidic biosensing.

An interesting combination for protein analysis is the structure proposed in [321], which combines an MZI and a hybrid plasmonic waveguide (HPWG) with nano-slots. Simultaneous use of two types of modes with TE and TM polarizations makes it possible to determine the optogeometrical properties (density and thickness) of protein layers. The authors have theoretically demonstrated this by analyzing the conformational change of HepV, a recombinant fragment of collagen V, during complicated molecular interactions. 

In [324], a sensor platform based on SiN nanophotonics was integrated with a microfluidic cartridge. The photonic chip consisted of six sensors. Each sensor contained an MZI, one arm of which interacted with the analyte through an evanescent field, and arrayed waveguide grating (AWG). AWG acts as a set of spectral filters that generate discrete output data from which the original MZI spectrum was further reconstructed. A change in n analyte leads to a shift in the peak in the transmission spectrum of the interferometer, and the concentration of substances can be determined. An LOD of 6 × 10^−6^ RIU and 19.478 ng/mL for C-reactive protein was obtained. Using the proposed platform reduces the cost of the entire device, since inexpensive detectors and broadband light sources (LEDs) can be used.

Table 7 also lists additional examples of resonant WG structures from recent publications.

##### 3.8.2.3. ARROWs

However, based on TIR, WGs often cannot be used because the analyte RI can be less than the surrounding wall’s RI, for example, for aqueous solutions (n_H_2_O_ = 1.33 versus typical values of n_walls_ = 1.4–3.5) [355]. An unusual way to solve this problem is a jet WG, i.e., a liquid jet in an air environment, for which TIR is performed at any n of liquid. This approach was demonstrated in [356] using a Cy5 aqueous solution.

A promising approach is the use of liquid core antiresonant reflecting optical waveguides (ARROWs), in which the formation of the WM is provided by multiple reflections from the layered cladding (specially selected transverse n profile, Figure 11n) of the WG due to the interference between the reflected and refracted rays. Reflectivity up to 99% can be obtained with four layers of cladding [357]. This allows for the creation of a hollow core that can be filled with a gas or liquid with the desired n. SiO_2_, Si_3_N_4_, Ta_2_O_5_ [358], TiO_2_ [359] and other materials in silicon substrate are used as layer materials. And a hybrid silicon-poly(dimethysiloxane) (PDMS) liquid core ARROW (h-ARROW) is also created [360]. Hybrid chips are convenient because the PDMS top layer is transparent and allows a wider range of optical detection methods to be used.

Fluorescence detection methods of single biomolecules have been developed in which exciting radiation is delivered to the microfluidic channel through solid-core ARROWs [361,362], as well as through multimode interference (MMI) WGs [363], which form the certain interference patterns along liquid core ARROWs. The authors of [325] have demonstrated the detection of SARS-CoV-2 and influenza A antigens with a target concentration of 30 ng/mL for the MMI-ARROW structure. The spectral multiplexing technique in MMI is described in [358]. Detection with a time-dependent fluorescence signal without the need for spectral demultiplexing upon detection is proposed. A similar dual-channel chip for detecting SARS-CoV-2 RNA and N protein was simultaneously demonstrated in [326], showing a LOD of 0.7 ng/mL for SARS-CoV-2 antigens.

However, the use of ARROWs is possible not only in a planar design. An approach with a combination of microfluidic channel and optical fibers, for example, hollow core fibers (HCF), is described. One study [327] demonstrates lab-in-fibers biological sensing technology. In HCF, containing eight hollow cladding channels, two channels are filled with liquid and can be described as liquid core ARROWs. One channel is used for interferon-gamma (IFN-γ) detection, the other is filled with NaCl solution and is used for temperature compensation, which is extremely important for the operation of the biosensor outside the laboratory (PoC application). For microfluidic channels, resonance minima are formed in the transmission spectra which depend on the refractive index of the analyte. Due to close thermooptical coefficients of liquids, a change in temperature leads to a comparable shift of the minima, and this can be used to compensate for temperature changes. The sensor has demonstrated a good sensitivity (LOD of 0.5 ng/mL).

#### 3.8.3. Other Micron- and Submicron Scale Structures

Various types of structures such as photonic crystals, metamaterials, subwavelength apertures and others are used in microfluidics. Zero-Mode Waveguides (ZMWs) or subwavelength apertures allow for the detecting of the fluorescence of single molecules and can be used to detect proteins and study protein-protein interaction [364], both when it is labeling [365] and in a label-free version (for example, when detecting its natural UV autofluorescence [366]).

In [328], an integrated into a microfluidic chip sensor based on gradient waveguide thickness guided-mode resonance (GWT-GMR) is proposed. The GWT-GMR sensor is a planar wedge-shaped Bragg grating waveguide. The structure has resonant properties. In the optical detection scheme, the structure is located orthogonal to the incident radiation which, under certain conditions, excites WGs that are losses. In this configuration, the GWT-GMR sensor works as a bandstop filter whose resonant wavelength depends on the analyte RI. The transmission intensity distributions on a CCD camera displays spectral information. The LOD of 2.92 g/mL for the concentration range of 0.8–500 g/mL for albumin, and 12.05 g/mL for the concentration range of 1–10,000 g/mL for creatinine is achieved.

In [322], a micromachined Fabry-Perot interferometer (µFPI) that consisted of two parallel and flat gold-coated mirrors, one of which had sub-wavelength nano-hole arrays, was proposed. Extraordinary optical transmission (EOT)-modulated surface plasmonic resonance (SPR) was used for RI measurement, with a sensitivity of 593 nm/RIU and a Q factor up to 128.4. By applying dielectrophoresis (DEP), a detection sensitivity of BSA protein enhancement of ~ 6-fold for 1 pM compared to 100 pM was realized.

Photonic crystal beads (PCBs) in 3D microfluidic chips for multiplex protein detection was used [330]. Three types of PCBs were made by a droplet template method from silica nanoparticles. PCBs were immobilized with capture antibodies and placed inside the chip for color coding for human immunoglobulin G (IgG), carcinoembryonic antigen (CEA), and anti-human alpha fetoprotein (AFP) detection. The authors of the work adapted the sandwiched fluorescence immunoassay technique for the investigated microfluidic chip. The target abundances were analyzed by the PCBs’ flourescence intensity. An LOD of 18.92 ng mL for AFP was obtained.

In general, the integration of optical elements and structures into a microfluidic chip usually requires optimization of element losses and the combination of technologies and materials in one planar chip. Also, the issue of temperature cross-sensitivity usually remains without discussion, although it is extremely important for PoC applications. Protein multiplex analysis is an important direction in the further development of micro- and optofluidic devices.

## 4. Impedance Spectroscopy Microfluidic Techniques and Methods for Proteins Detection

### 4.1. Electrical Impedance Spectroscopy

Electrical impedance spectroscopy (EIS) makes it possible to define the dielectric properties (complex dielectric constant values *ε*′ and *ε*″) of a medium as a function of frequency. The list of recently published medical EIS works far from being a complete one is [367,368,369,370,371,372,373]. EIS is based on the interaction of an external electrical field with the electric dipole moment of the medium and therefore the frequency response of the system, including the energy storage and dissipation properties, is revealed. The frequency range of impedance spectrometry is extremely wide (from units of kHz to hundreds of GHz), however, we will consider the works of various medical diagnostics of the radio frequency (RF) and microwave range (MW). 

The permittivity is related to the extent to which charged particles can be displaced or polarized under the influence of the electric field. The dielectric constant *ε* is a complex quantity:

(3) $$\varepsilon^{*}\left(\omega \right)={\left(\omega \right)-i\varepsilon \left(\omega \right)}^{\prime \prime },$$

where *ε*′ is the dielectric constant of the medium, proportional to the change in the free energy accumulated by the medium during the period of the field oscillation, and *ε*″ is the factor proportional to the energy absorbed during the period of the field oscillations. 

The ratio of the imaginary part of the complex permittivity to its real part is called the tangent of the loss angle:

tanδ = *ε*″/*ε*′, (4)

where δ is the angle that complements the phase shift between the applied voltage and the current through the dielectric to π/2.

The dielectric permittivity characterizes how charged particles can be displaced or polarized under the influence of the electric field. Each polarizable entity within the tissue will exhibit its own characteristic response and thus a distribution of relative permittivity will give rise to a complex function of frequency in the form of Debye expression:

(5) $$\;\varepsilon^{*}=\varepsilon_{\infty }+\frac{\varepsilon_{s}-\varepsilon_{\infty }}{1+i\omega \tau }$$

where *ε*_∞_ is the high frequency permittivity at which the polarizable entities are unable to respond, *ε_s_* is the low frequency permittivity where polarization is maximal, *ω* is the angular frequency, and *τ* is the characteristic relaxation time of the tissue under study. A dielectric dispersion is therefore associated with biological tissues in which the relative permittivity decreases with increasing frequency [374]. In general, three frequency ranges of dispersion namely α β, γ can be identified in biological tissues and in particular for protein: α corresponds frequencies up to few kHz, β-frequencies up to f~10^7^ Hz and γ range ≥ 10^9^ Hz [374].

Selecting the real and imaginary parts in expression (5),

(6) $$\varepsilon^{\prime }=\varepsilon_{\infty }+\frac{\varepsilon -\varepsilon_{\infty }}{1+\omega^{2}\tau^{2}}$$

(7) $$\varepsilon^{\prime \prime }=\frac{\left(\varepsilon -\varepsilon_{\infty }\right)\omega \tau }{1+\omega^{2}\tau^{2}}$$

Equation (7) shows that the imagery part of the dielectric constant tends to zero at both small and large values of *ωτ* and reaches a maximum at $\omega_{m}\tau =1$.

Experiments show that the Debye equations are applicable for the relaxation process of polar molecules in infinitely diluted solutions of polar liquids in non-polar solvents if the molecules of polar liquids are large compared to the molecules of the solvent. These equations are also applicable to polar liquids, the molecules of which are almost spherical and for which relaxation phenomena are characterized by a single relaxation time. However, most polar liquids have a wide dispersion region with several relaxation times, and the Debye equations are not directly applicable to describe dispersion in such cases. For the case when there is a distribution of relaxation times, Cole and Cole [375] proposed the following empirical formula instead of the Debye equation:

(8) $$\varepsilon^{*}=\frac{\varepsilon -\varepsilon_{\infty }}{1+{\left(i\omega \tau \right)}^{1-\alpha }}+\varepsilon_{\infty }$$

where α is called the coefficient of distribution of relaxation times, and (0 < α < 1), *τ*_0_ is the most probable value of the relaxation time.

Determination of dielectric parameters of a medium is based on measurements of its electrodynamic characteristics using a frequency-dependent relationship between the total complex impedance (*Z*), conductivity (*σ*) and relative permittivity (*ε_r_*) [374]:

(9) $$Z=Z^{\prime }+j\omega =1/\left(\sigma +j\omega \varepsilon_{0}\varepsilon_{r}\right),$$

where *Z*′ and *Z*″ are the real and imaginary components, *ω* is the radial frequency, and *ε_o_* is the permittivity of free space. Both *Z*′ and *Z*″ can be, measured, from which the conductivity and relative permittivity are defined. The results of *Z* measurements of transmitted lines or resonators filled liquid are carried out on the basis of data on the level of reflected (S11) and transmitted (S21) signals. These values can be converted to complex dielectric constant values (*ε*′ and *ε*″). 

Dielectric spectra of aqueous protein solutions are well studied and show at least three dispersion regions, which are often termed β-, γ- and δ-relaxations [376]. The β-relaxation in the low frequency range can be assigned to the rotation of the polar protein molecule in its aqueous medium; δ-dispersion can be explained by a bound water relaxation or additional effects such as intra-protein motions [377]; the γ-relaxation at around 18 GHz (at room temperature) is the reorientational motion of the free water molecules (similar to the main relaxation process in pure water) [376].

Examples of the applying of EIS for protein detection in recent years are shown in Table 8.

Structurally, microfluidic devices for electrical impedance spectroscopy are capacitors (plane-parallel or planar) integrated into microfluidic channels. The electrodes of such capacitors are isolated from the liquid by thin dielectric layers. Microfluidic impedance sensors can be either disposable (more often) or reusable (less often), depending on the materials from which they are made. With increasing frequency, the sensor design may become more complicated, particularly requiring the use of microwave resonators in the GHz range.

Examples of using microfluidic sensors for relatively low frequencies can be found in [80,378,379]. In [80], a microfluidic impedance spectroscopy sensor is shown which is able to characterize protein solutions in a wide frequency range corresponding to the β and δ regions of dielectric dispersion. In this work, the authors describe the technology of the sensor manufacturing process and report on the experimental results of monitoring a solution of bovine serum albumin protein in phosphate-buffered saline. In [378], the authors evaluated the ClotChip, which was a three-dimensional capacitive sensor with parallel plates integrated into a disposable microfluidic channel with a minimum sample volume (<10 µL), used for assessment of whole blood coagulation. The ClotChip reading was defined as a temporary change in the actual part of the dielectric constant of whole blood at a frequency of 1 MHz. The authors [378] conclude that ClotChip evaluates many aspects of the haemostatic process in whole blood on a single disposable cartridge, which highlights its potential as a POC platform for rapid and comprehensive haemostatic analysis. In [379], flexible hybrid paper-plastic microchips with composite electrodes made of silver and graphene were developed for rapid and selective diagnostic immunoassay for the determination of alpha-fetoproteins. The system works on the basis of measuring changes in the impedance of electrodes printed on a paper microchip which are associated with changes in protein concentrations. The authors of this work have shown that their concept of peptide modified paper chips allows for the screening of alpha-fetoproteins in a wide clinical range (from 1 to 104 ng/mL) in human serum in the detection zone of modified microchips with a detection limit of 10 ng/mL.

Examples of the design and use of microfluidic sensors in the microwave range can be found in the works of [380,381,382]. In [380], a microwave method and a dielectric resonator-microfluidic system for non-destructive determination of hemoglobin concentration in microliter blood samples are described, where a connection between the dielectric properties of mouse blood and the concentration of hemoglobin is established using broadband microwave spectroscopy (from 200 MHz to 40 GHz). For the samples with volumes less than 10 μL, the limit of detection was around 0.34 g/dL. In [381], the study’s authors demonstrated a symmetrical split ring resonator-based microwave sensor with spurline filters for detecting and characterizing the properties of drugs. In [382], they used an interdigitated electrode sensor and showed that it is capable of detecting various concentrations of albumin (from 0 to 100 g/L) with a high degree of repeatability at 200 MHz and 4 GHz.

### 4.2. Electrochemical Impedance Spectroscopy

Electrochemical impedance spectroscopy is another type of impedance measurement, and requires the electrolyte, which is a material under test/biological fluid and two non-isolated “interdigitated electrodes” (IDEs) [383] formed from different metals or 3 non-isolated electrodes: reference electrode (Ag/AgCl or KCl), platinum counter electrode and the working electrode (usually it is gold with the functionalization layer for biosensors) [384]. 

To describe the working principle one can assume that a DC polarization potential is applied, e.g., 0.2 V. The impedance can be determined by applying small-signal perturbations at frequencies typically below 100 kHz, e.g., of 5 mV sine wave of approximately a 0.2 V common-mode potential (resulting in voltage levels of 0.195 V to 0.205 V). During this time only non-Faradic current will flow. The applying of the alternating current in the form of sine-wave oscillations will prevent the reorganizations of the ions in the solutions, as the voltage changes are faster. Next, the frequency can be experimentally decreased and therefore the kinetic and mass transfer control of the system will be investigated. 

**Figure 12:**
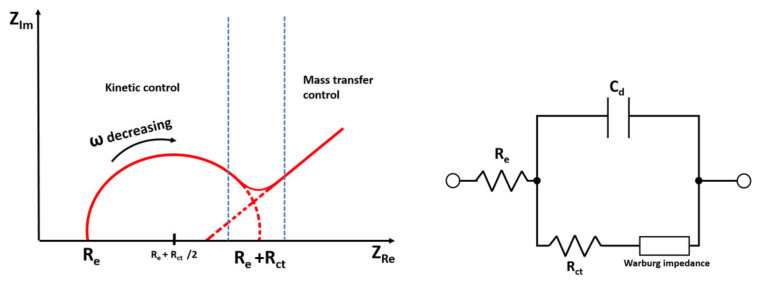
Depiction of the typical impedance spectrum (red line, dependance of the real Z_Re_ and imaginary Z_Im_ parts of impedance) and equivalent circuit. C_d_ is an interface electrode-fluid capacitance, R_e_–solution resistance, R_ct_–charge transfer or polarization resistance, ω–perturbation frequency; Warburg impedance models the diffusion behavior of impedance.

Figure 12 shows a typical impedance spectrum, and it starts from R_e,_ which is zero at very high frequencies. Therefore, mass transfer does not take place. With the decrease of the perturbation frequency, capacitive properties of the liquid-electrode interface start to play a role, and impedance rises. In the next region, when the impedance depends on the diffusion of reactants towards or away from the surface, the Warburg impedance starts to play a larger role. It rises from mass transport and has a particular low frequency character. However, if we neglect the Warburg impedance and continue the circular line to the *x*-axis, we will come to the R_e_ + R_ct_ (solution resistance + charge transfer or polarization resistance) region. This exact part of the spectrum is extremely important for the electrochemical impedance spectroscopy with the functionalized working electrode surface.

**Figure 13:**
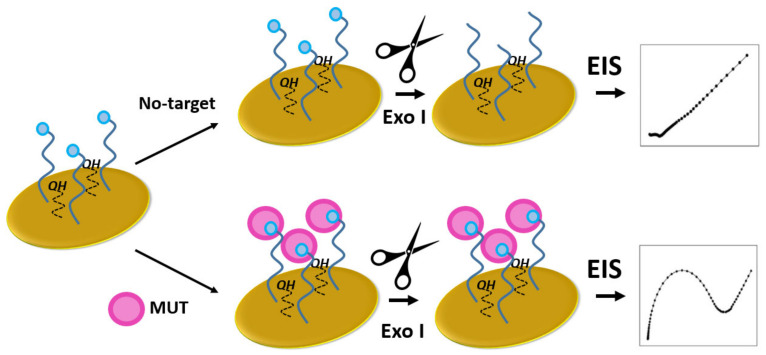
Schematic illustration of the label-free electrochemical impedance biosensor for protein detection based on terminal protection by hydrolysis with exonuclease I (Exo I) of small molecular linked-DNA (adapted from [384]).

For the sensing applications, two different situations can arise: the impedance of the biological material is considered as a function of the material under test (MUT) concentration, or the biological component is immobilized on the working electrode and the interaction with an analyte molecule is detected (Figure 13). The second option is widely used in the biosensor field. The functionalization of the working electrode surface with antibodies or another special reactive solution enable the selectivity of such a sensor. The schematic of this principle is shown in Figure 13 [384]. In this work, the authors created a label-free electrochemical impedance biosensor for protein detection based on the terminal protection of small molecule-linked DNA. The material under test was folate receptor (FR), which is binding to the folate (FA). The detection limit performed by this method remains 3 pM, which proves the high sensitivity of this approach. Thus, Scharma et. al, achieved the detection limit of 90 fg/mL and time-to-result of 15 min for human interleukin-8 protein in serum by developing the electrochemical impedance-based biosensor [385]. 

**Figure 14:**
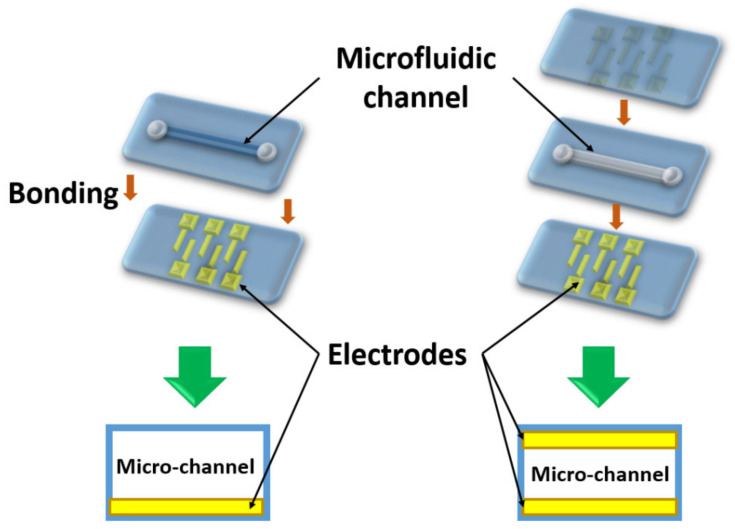
Schematic view on the possible electrodes location in a microfluidic chip. The left–microfluidic channel is structured in the layer, the right–channel is cut through the layer and one more slide with electrodes on top. The black arrows point the location of the microfluidic channel and electrodes, red arrows are leading through the technological process and green arrows point the resulting structures.

Electrochemical impedance spectroscopy is a label-free detection method which allows a high sensitivity and selectivity of protein detection. Furthermore, this method is applicable to microfluidics. The electrodes implementation into the microfluidic channel is no longer a challenge. Often the chip consists of two parts: the first part is glass or silicone with golden electrodes sputtered on top of it, and the second part is bonded on top of a molded or hot-embossed polymer microfluidic structure. Several groups achieved good results with this approach using microfluidics [99,386,387,388]. By using two glass layers with electrodes on the top and bottom, with a microfluidic channel cut through an additional sandwich layer in between, it is possible to fabricate sensor electrodes placed on two opposite sides (Figure 14). Both configurations are suitable for protein detection and other biomedical applications [389]. 

Examples of the applying of electrochemical impedance spectroscopy for protein detection in recent years are shown in Table 8.

**Table 8:** Some examples of the applying of impedance methods for protein detection in recent years.

Structure	Detection Principle/Notes	Target Analyte	Limit of Detection (LOD) // Sensitivity	[Ref.]/Year
Flexible platinum electrodes	Voltammetric measurements, Electrochemical impedance spectroscopy	Dopamine, Parkinson’s disease protein 7	5.1 × 10^−6^ mol/L, 7.5 ng/mL // −	[390] 2020
Capacitive sensor in a microfluidic channel	Dielectric spectroscopy 10 kHz to 100 MHz/sample volume < 10 μL	Blood coagulation factor, platelets	−	[378] 2018
Microwave dielectric resonator–microfluidic system	Broadband microwave spectroscopy 200 MHz and 40 GHz/sample volume < 10 μL	Hemoglobin	SD ≈ 0.34 g/dL	[380] 2016
Microfluidic impedance biosensor	Electrochemical impedance spectroscopy	Troponin I	1 ng/mL // −	[63] 2021
Microfluidic impedance biosensor	Electrochemical Impedance Spectroscopy	Prostate Specific Antigen	1 ng/mL // −	[391] 2013
Biomimetic sensors	Electrochemical Impedance Spectroscopy/Human serum analysis	Adiponectin, Leptin	0.25 μg/mL, 0.110 ng/mL // −	[392] 2020
Paper microfluidic biosensor	Electrochemical Impedance Spectroscopy/Functionalized multi-walled carbon nanotubes	Troponin I	0.05 ng/mL // 1.85 mΩ/ng/mL	[393] 2019
Multiwell microelectrode array	Electrochemical Impedance Spectroscopy	Tau protein	–	[394] 2016
Microfluidic immunosensor	Electrochemical Impedance Spectroscopy/Polyethylenimine coated graphene electrode, wide dynamic range 1 pg/mL to 100 ng/mL	Glial fibrillary acidic protein	1 pg/mL // −	[395] 2018
Disposable microfluidic amperometric dual-sensor	Electrocatalytic reduction/Human blood analysis	Glycated hemoglobin, total hemoglobin	3.7 nM, 82 nM // −	[396] 2017
Microfluidic immunosensor	Pulse voltammetry, Electrochemical impedance spectroscopy	Epidermal growth factor receptor 2	1.0 fM, 1.0 pM // 0.585 μA/μM × cm^2^, 43.7 kΩ/μM × cm^2^	[397] 2016
Plastic-paper microfluidic chip	Impedance spectroscopy in the frequency range of 100 Hz to 100 kHz/Human serum analysis	Alpha-fetoprotein	10 ng/mL // −	[379] 2018
Molecular imprinted polymer (MIP)-based impedimetric sensor	Electrochemical impedance spectroscopy	NS1 (non-structural protein 1—a specific and sensitive biomarker for dengue virus infection)	0.3 ng/mL	[398] 2020
Symmetrical split ring resonator (SSRR) based microwave sensor	Microwave spectroscopy	Drugs	−	[381] 2017
Interdigitated electrode sensor	Microwave spectroscopy/cerebrospinal fluid analysis, wide dynamic range 0 to 100 g/L	Albumin	−	[382] 2015

## 5. Conclusions

The article reviewed the physical methods for detection, quantification, and characterization of proteins in microfluidic platforms. For that reason, the choice of detection principles was based on electromagnetic waves, as the direct interaction with protein macromolecules in solutions can serve as a basis for their label-free characterization. Label-free techniques are less often used because of still relatively low sensitivity and specificity compared to labelling approaches, but despite obvious limitations they allow breakthrough in affordability, avoid sample preparation, and reduce analysis times. Moreover, label free detection approaches hold great potential to realize a unique capability of protein analysis in their native conformational state. Technological progress in the field of microfluidics has made it possible to move to a new level of biosensor design and now label-free systems present a good alternative solution to conventional analytical platforms.

The main aim of this work was to review label-free protein techniques based on various physical phenomena as a broad but separate and distinct class of analytical methods in proteomic science. Undoubtedly, optical methods play a leading role among other label-free protein techniques because of non-invasiveness and easy integration with microfluidic structures made of quartz, glass, PDMS or other optically transparent materials. 

UV absorption spectroscopy and native fluorescence are the most well-established techniques, and have been used for decades. Despite fundamental limitations, it is still too early to dismiss them because they do not require sophisticated equipment and still can be a method of choice in protein biosensors, especially when high sensitivity and specificity are not necessary. Moreover, multireflection optical cells, optical elements directly integrated in a microfluidic structure, and sensing microfibers with evanescent field absorption, which have been recently introduced in microfluidic devices, can sufficiently improve the capabilities of these techniques. 

ATR FTIR spectroscopy and other IR techniques including surface-enhanced infrared absorption spectroscopy, infrared reflection absorption spectroscopy, vibrational circular dichroism, and microfluidic modulation spectroscopy provide an indispensable tool for the structural analysis of proteins, the investigation of molecular interactions, the detection of protein molecules in a native conformational state, and chemical imaging in microfluidic chips. IR spectroscopy is more suitable for bench research than for biomedical diagnostics, but recent advances in portable and robust FTIR spectral equipment made it more affordable and easier to use even outside of the laboratory environment.

Detection methods and optical biosensors based on surface plasmon resonance, including various implementations of SPR, LSPR, and SERS, are the most sensitive among other label-free techniques for protein biomarker detection. It can be safely said that plasmonic biosensors are the future of clinical diagnostics because they enable detection of very small amounts of analyte, even single molecules. For example, such biosensors can discover miniscule concentrations of cancer biomarkers (proteins, cells, DNA) which are found in bodily fluids such as blood or urine. It is believed that this approach, often called a liquid biopsy, could eventually substitute, or at least compliment, traditional surgical biopsy.

Optofluidics is an emerging trend in optical biosensor technology. It combines the advantages of microoptics and microfluidics; the integration of optical elements into a microfluidic chip allows one to implement optical methods for detecting and analysis of biomolecules. The advantages of the approach include miniaturization, the ability to receive a signal from small volumes of analyte, a decrease in detection thresholds; and increasing of the sensitivity. Optofluidic devices can often achieve better characteristics compared with conventional off-chip optical elements using the same analytical method.

Broadband electrical impedance spectroscopy is a widely used and relatively simple tool for studying the dynamics of protein solutions in microfluidic structures. The development of the method is not only in the technical and technological plane but also presupposes an improvement in the interpretation of measurement results, which presuppose a description of the molecular nature of protein dipoles, a quasi-macroscopic subensemble of interfacial waters producing interfacial polarization, as well as the molecular dynamics of distinct protein conformations. Electrochemical impedance spectroscopy is a fairly sensitive electrochemical method for label-free monitoring of bio-recognition events on the electrode surface. Due to this, it provides a low detection limit. Detection is fast, with a short detection time. An important problem remains the ability to transform such capabilities from pure buffer solutions of the research environment into more practical conditions and real clinical samples; furthermore, a number of recent studies have revealed noticeable progress in this direction. Impedance spectroscopy, both electrical and electrochemical, is extremely important for biosensing and biomarker detection, including cancer diagnostics. 

The current review contributes to the field of biomedical research and grasp over the possibilities and limitations of these techniques. The review will support the development of an appropriate physical method or a combination of several methods for solving particular tasks. The authors believe that the article will be particularly useful for the specialists in the fields of microfluidics and POC diagnostic devices in medicine, biology, drug discovery, and food analysis, etc

## Figures and Tables

**Table 1:** Protein absorption coefficients in the UV region in 1 g/L water solutions.

Protein	Absorption Coefficient, 1/cm	
	280 nm	205 nm
Human serum albumin	0.59 [153]	32.7/30.5 (fraction II/IV) [154]
Bovine serum albumin	0.66 [155]	29.6 [153]
Human IgG (serum)	1.33 [156]	
Human IgA (serum)	1.34 [156]	
Human IgM	1.33 [156]	
Human myoglobin	1.74 [157]	
Insulin	1.05 [158]	38.1 [154]
Fibrinogen	1.56 [159]	
β2-microglobuline (human urine)	1.70 [159]	
Human α-lactoalbumine	1.53 [156]	
Chicken lysozyme	2.65 [159]	35.4 [155]

**Table 2:** Aromatic amino acids absorption and fluorescence [160,161].

Amino Acid	Optical Properties				
	Absorption		Fluorescence		
	λmax, nm	$\mathrm{Molar}\;\mathrm{Extinction}\;\mathrm{Coefficient},\;\;\frac{1}{\mathrm{cm}\times \mathrm{mol}}$	Fluorescence Lifetime, ns	λmax, nm	Quantum Yield
Tryptophane	280	5600	3.1	348	0.2
Tyrosine	274	1400	3.6	303	0.14
Phenylalanine	257	200	3.4	272	0.04

**Table 3:** Vibrational absorption bands of proteins in the MIR region.

Absorption Band	Wavenumber, cm^−1^	Description
Amide I	1600–1690 [163]	C=O stretching
Amide II	1480–1575 [163]	CN stretching, NH bending
Amide III	1229–1301 [163]	CN stretching, NH bending
Arginine	1652–1695 [163]	νa_s_(CN_3_H_5_^+^ )
Glutamine	1556–1560 [163]	νa_s_(COO−)
Tyrosine	1498–1500 [162]	ν(CC), δ(CH)
Tyrosine	1269–1273 [162]	ν(C–O), ν(CC)

**Table 4:** Applications of IR spectroscopy in microfluidic devices for investigation of proteins.

Spectroscopic Technique	Target Proteins	Characterization	Reference
MMS	Therapeutic monoclonal antibodies (mAb)	Secondary Structure Analysis	[193]
SEIRAS with plasmonic nanoantennas	Alpha-synuclein	Secondary structure	[194]
Derivative ATR FTIR spectroscopy	Granulocyte colony-stimulating factor (rhG-CSF)	Secondary Structure	[195]
TM-FTIR	Hemoglobin (H2500), poly-l-lysine (P2636)	Protein-Conformation Studies	[196]
Time-resolved FTIR	Ubiquitin	Protein folding	[197]
ATR FTIR	Alzheimer’s b-amyloid	Secondary structure	[198]
FTIR difference spectroscopy	Myoglobin	CO photodissociation	[199]
SEIRAS	Cytochrome c	Monolayer structure morphology	[200]
SEIRAS	Cytochrome c	Functionality of a Protein Monolayer	[201]
SEIRAS	α-synuclein	Lipid–protein interactions	[202]
SEIRAS	Tripeptide glutathione (GSH)	Ultrasensitive detection	[203]

**Table 5:** Application of label-free fluorescence for detection of proteins in microfluidic devices.

Spectrosocopic Technique/Notes	Target Protein	LOD	Reference
Intrinsic protein fluorescence, conformational changes UV LED excitation at 295 nm, detection at 330 nm	tryptophan, bovine serum albumin (BSA), bovine carbonic anhydrase (BCA)	72 nM 128 nM 250 nM	[216]
Intrinsic protein fluorescence, visualization UV LED excitation at 280 nm	BSA	500 nM	[217]
Intrinsic protein fluorescence, qualitative determination UV LED excitation at 280 nm	troponin T	6.5 ng/mL	[218]
Intrinsic protein fluorescence, detection for microchip electrophoresis Laser excitation at 266 nm detection with PMT based spectrometer	lysozyme, trypsinogen, chymotrypsinogen conalbumin, ovalbumin	12.5 μg/mL	[219]
Intrinsic protein fluorescence, continuous electrophoretic separation via free flow isoelectric focusing (FFIEF) Laser excitation at 266 nm	α-Lactalbumin, β-Lactoglobulin B, Albumin, Globulins	300 μmol/L	[220]
Intrinsic protein fluorescence, electrophoresis visualization, two-dimensional fingerprinting UV LED excitation at 280 nm	BSA, human lysozyme	100 nM	[221]
Two photon excited (TPE) fluorescence laser excitation at 420 nmdetection at 320 nm	tryptophan lysozyme, trypsinogen and chymotrypsinogen	12.5 ug/mL	[222]
Fluorescence lifetime detection, microchip electrophoresis laser excitation at 266 nm	lysozyme, trypsinogen and chymotrypsinogen	2.5 mg/L	[223]
Fluorescence Correlation Spectroscopy laser excitation at 266 nm detection in 310−410 nm	β-galactosidase streptavidin penicillin amidase	-	[224]
Förster resonance energy transfer (FRET) Excitation at 280 nm, detection at 350 nm	Albumin	0.15 nM	[225]

**Table 6:** Application of label-free SERS for detection of proteins in microfluidic devices.

Target Protein	Pathological Condition	Reference
Human serum albumin	different stage liver cancer	[284]
Erythropoietin isophorms	anemia in cancer patients, athletes	[285]
Serum proteins	breast cancer	[286]
Prion proteins	Creutzfeldt-Jakob disease, kuru, fatal familial insomnia, and Gerstmann−Sträussler−Scheinker (GSS)	[287]
Phosphorylated proteins (Tau Biomarkers)	Alzheimer’s disease	[288]
Saliva proteins	oral cancer	[289]
Insulin	diabetes, hyperinsulinemia	[290]
Immune checkpoints proteins	cancer	[291]
Single cell metabolites	cancer	[292]
Ovarian Cancer Antigen CA125	ovarian Cancer	[293]
Myoglobin	radiation-induced injury	[294]
Serum proteins	colorectal cancer	[295]
Interleukins	immunological disoderes	[281]
90 K biomarker	cancer	[282]
Thrombin	blood coagulation	[263]

**Table 7:** Some examples of the applying of integrated optical elements in microfluidics for protein detection in recent years.

Optical Component or Structure	Detection Principle/Notes	Target Analyte	Label-Free	Limit of Detection (LOD) // Sensitivity	[Ref.], Year
Optofluidic laser TIIA (OFL-TIIA)	Dependence of the laser emission intensity on the IgG concentration in the RhB solution inside a Fabry-Pérot cavity/Wide dynamic range (1.8 × 10^−10^–1.8 × 10^−5^ g/L)	Rabbit IgG	No	1.8 × 10^−10^ g/L // -	[307] 2019
2D microlenses, mirrors and optical fibers	Absorbance in six channels/Parallel measurements at different optical lengths (MPHIL concept)	Proteins: HEWL, GI, BLL, FASE, DHP	Yes	From 1.28 ± 0.04 μM to 8 ± 2 μM for different channel for the GI // -	[308] 2015
Array of dielectric microspheres	Fluorescence of functionalized Au NPs enhanced by PNjs from microspheres	Biotin and mouse IgG	No	Fluorescence intensity was enhanced by a factor ∼40	[309] 2015
Array of dielectric microspheres	Fluorescence (FCS method)/Increasing detection volumes up to several tens of femtoliters	Protein Annexin A5b	No	Concentrations in the picomolar range	[310] 2014
Ge on Si (GOS) WG	MIR spectroscopy/Measurement of aqueous protein	BSA protein	Yes	Chip was tested with 900 µM BSA solution	[311] 2020
Liquid-core WG modified with gold NPs	SERS with enhancement factor of 2.7 × 10^8^ for R6G	R6G, BSA protein	Yes	10^−11^ mol/L (R6G) // - Chip was tested with 10^−5^ mol/L of BSA	[312] 2017
FLOW	Wavelength shift of the transmission spectrum in the optical fiber/Log–linear response at concentrations ranging from 10 fg/mL up to 10 ng/mL	Protein p53	Yes	10 fg/mL // 22.2 pm/(fg/mL)	[313] 2018
Slot WG with grating	Wavelength shift of resonance in grating for different analyte n	hemoglobin, globulin and BSA protein	Yes	- // 600 nm/RIU (300 FOM)	[314] 2019–2021
OMNFs	LSPR (gold NPs on the fiber surface)	Streptavidin	Yes	1 pg/mL // -	[315] 2018
Diffraction-based leaky waveguides (LWGs)	RI sensing (Defining resonances in reflectivity curves)/Chitosan WGs	BSA protein	Yes	1.9 × 10^−6^ ± 1.3 × 10^−6^ RIU // 125.5 ± 3.8 deg/RIU	[316] 2021
Array of nanomaterials coated FBGs	Bragg wavelength shift/Multiparameter sensing (pH, temperature, humidity, gas concentration, light intensity and protein concentration)	BSA protein	Yes	- // 5 pm/μg/mL	[317] 2015
Plasmonic TFBG sensor	RI sensing; differential amplitude measurement between the plasmonic and cut-off resonances/Minimal temperature cross-sensitivity	Rat urinary protein	Yes	10^−5^ RIU; 1.5 × 10^−3^ mg/mL // 8000 dB/RIU; 5.5 dB/(mg/mL)	[318] 2016
LPG coated with graphene oxide	Wavelength shift of the resonance in a transmission spectrum/Wide dynamic range (1 ng/mL–100 μg/mL)	C-reactive protein	Yes	0.15 ng/mL // -	[319] 2021
PC cavity and WG	Resonance wavelength shift/Q = (1.2–2.2) × 10^4^	Protein markers: fasligand (f), chemokine ligand 4 (MIP1) and hepatic growth factor (HGF)	Yes	9.813 pg/mL (HGF); 15.437 pg/mL (MIP1); 0.3346 pg/mL (f) // 68–112 nm/RIU	[320] 2020
MZI + hybrid plasmonic waveguide (HPWG) + nano-slots	Change in the dip (resonance) depth in the transmission spectrum at fixed wavelengths—amplitude measurements/Bulk RI measurement; biosensor are designed and theoretically investigated	HepV	Yes	Output transmission spectra and methodology for calculating parameters were reported	[321] 2017
µFPI with sub-wavelength nano-hole arrays	Change in EOT-modulated SPR patterns/Q factor up to 128.4	BSA protein	Yes	1 pM // 593 nm/RIU	[322] 2019
Array of six asymmetric MZI (aMZI)	Measuring the phase shift of the output signal (RI sensing)	Periostin (POSTN) and transforming growth factor beta-induced protein (TGFBI)	Yes	16 × 10^−8^ RIU; 10 ng/mL // ≤5000 nm/RIU	[323] 2021
MZI and AWG	Spectral shift (RI sensing)/Low-cost instrumentation	C-reactive protein	Yes	6 × 10^−6^ RIU; 19.478 ng/mL // -	[324] 2018
MMI-ARROW	Fluorescence excitation via MMI waveguides orthogonal to the microfluidic channel aligned with the ARROW, which traps the luminescence emission	SARS-CoV-2 and influenza A antigens	No	30 ng/mL // -	[325] 2021
multi-channel MMI-ARROW chip	The same/Dual detection of nucleic acid and antigen biomarkers with single molecule sensitivity	SARS-CoV-2 nucleic acids and proteins	No	0.7 ng/mL (SARS-CoV-2 antigens) // -	[326] 2021
2 ARROWs in HCF	Wavelength shift of two resonance/Temperature compensation	Interferon-gamma (IFN-γ)	Yes	0.5 ng/mL // 1413 nm/ RIU	[327] 2020
GWT-GMR sensor	Sensor converts spectral information to spatial information on a CCD (resonance band shift when n (biomolecule concentration) changes)	Albumin, creatinine	Yes	2.92 µg/mL (albumin); 12.05 µg/mL (creatinine) // 24,497 µm/RIU	[328] 2021
Subwavelength grating metamaterial (SGM) ring resonator	Wavelength shift of resonance in transmission spectra/Q~2180 at 1548.81 nm.	Streptavidin	Yes	10 μg/mL or ∼4.2 × 10^−4^ RIU // 310–423 nm/RIU	[329] 2021
Photonic crystal beads (PCBs)	Fluorescence immunoassays/Multiplex bioassays	Human IgG, AFP and CEA	No	25.33–105.45 ng/mL (IgG); 18.92 ng/mL (AFP) // -	[330] 2018

## Data Availability

Not applicable.
